# Generative artificial intelligence in animal genomics for smart agriculture: Applications, challenges, and future prospects

**DOI:** 10.1016/j.vas.2026.100702

**Published:** 2026-05-18

**Authors:** Navid Ghavi Hossein-Zadeh

**Affiliations:** Department of Animal Science, Faculty of Agricultural Sciences, University of Guilan, Rasht, 41635-1314, Iran

**Keywords:** Animal genomics, Digital twins, Generative AI, Large biological models, Precision livestock farming, Smart agriculture, Sustainable breeding and management

## Abstract

Generative artificial intelligence (AI) is becoming a groundbreaking paradigm in the field of animal genomics and is providing the possibility to take a step towards intelligent agriculture, with better data integration, predictive modeling, and biological design. This review focuses on the shift from predictive to generative modelling paradigms, examining their implications for data synthesis, biological sequence design, and integrative smart livestock systems. It provides a comprehensive overview of recent developments, applications, and challenges at the intersection of generative AI and animal genomics, as well as future directions. In doing so, it sheds light on novel opportunities and constraints specific to livestock genomics that are not adequately addressed in broader AI or human genomics studies. Thus, it bridges the gap between computational innovations and biological constraints. It initially sets the conceptual background in place by looking at the development of smart agriculture, the essentiality of animal genomics, and the development of generative model architectures in life sciences, as well as fundamental methodological aspects, including livestock genomic and multi-omics data peculiarities and the representation of biological sequences. The review then comprehensively discusses a wide range of applications such as genomic data augmentation, prediction of new genetic variants, design of protein and gene sequences, augmentation of genomic selection and trait prediction, regulatory and epigenomic modeling, accurate breeding and reproductive technologies, and cross-species genomic modeling, illustrating how generative AI is transforming genomics into something generative, enhanced through simulation. It is discussed in terms of integration into systems of smart agriculture, connections with precision livestock farming, digital twin, genomics-to-management pipelines, and sustainability-focused systems of decision-making, where the adaptive, individualized, and system-level optimization can be applied. Critical analysis of major challenges and limitations, such as heterogeneity and scarcity of data, model bias and generalization, computational and resource limitations, validation and interpretability issues, and ethical, legal, and social constraints that drove the responsible deployment are also critically reviewed. Lastly, the future opportunities are discussed, which should center on generative genome engineering, multimodal and federated modeling, species preservation, real-time interaction with smart farming technologies, and the creation of responsible and ethical AI frameworks. Overall, it is possible to state that this review makes generative AI a base technology of the new generation of animal genomics and smart agriculture, but it highlights that interdisciplinary cooperation, stringent validation, and alignment with the notions of sustainability, animal welfare, or values are necessary to realize its capabilities.

## Graphical abstract

**Figure:**
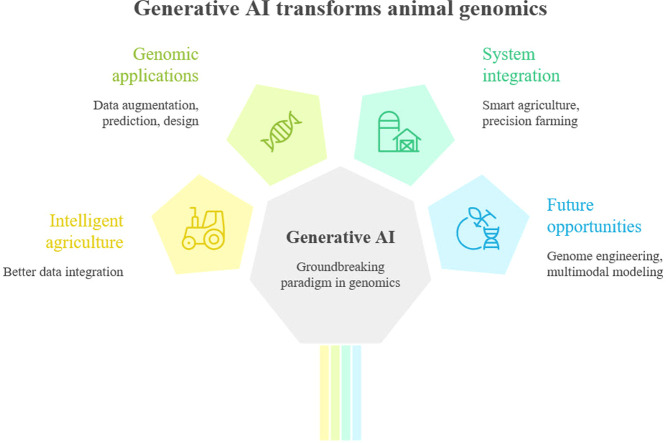

## 1 Introduction

The accelerating digitalization of the agricultural sector has led to the emergence of the concept of smart agriculture, which represents an adaptive, data-driven model aimed at meeting the increasing needs for productivity, sustainability, and resilience. Smart agriculture offers the opportunity to monitor environmental processes and manage them continuously by leveraging sensing technologies, automation, connectivity, and computational intelligence. This paradigm is a radical change in experience-based decision-making to systems, which make use of real-time data and predictive analytics to optimize results in times of uncertainty. With agricultural systems growing more complex as a result of climate variability, resource limitations, and social demands for sustainability and welfare, the concept of smart agriculture offers a model to manage the complexity in coordinated technological and biological innovation (Balyan et al., 2024; Sajib & Sayem, 2025; Ghavi Hossein-Zadeh, 2026).

Livestock production plays a pivotal role in smart agriculture since animal production systems have access to very complex types of interactions among the genetic aspects, physiology, environment, and management practices. Such interactions not only affect productivity and efficiency but also the health of animals, their welfare, and the viability of the long-term system. Livestock production can be conducted at many scales of time, in contrast to many other crop systems, where time is characterized by short-term physiological interactions. Therefore, efficient smart livestock solutions need to combine dynamic streams of data with fixed sources of biological data. In this respect, artificial intelligence (AI) has become a decisive enabling technology, serving as an aid in the interpretation of high-dimensional and heterogeneous data and adaptive management strategies that modify and react to the changing conditions and consider biological constraints (Ghavi Hossein-Zadeh, 2025; Zhu et al., 2025).

Animal genomics offers a basis of biological information, which is applied in smart agriculture to understand the genetic makeup of complex traits on a molecular scale. This is because the genomic information bases heritable variation and thus defines the potential of an animal to grow, reproduce, be healthy, and adaptive in the future, which is not affordable in the short run; it makes it valuable in long-range planning and thus strategic decision-making. Genomic data is a rather stable feature that is preserved across generations, unlike phenotypic observations, which are highly dependent on fleeting environmental conditions. Genomics application in smart agriculture thus facilitates management systems to shift to more proactive approaches to reactive ones in line with long-term genetic goals, enhancing sustainability of increase as opposed to single-handedly optimizing (Ghavi Hossein-Zadeh, 2024; Boktar, 2025).

Regardless of the potential, the smart utilization of animal genomics in agriculture poses significant analytical problems. Genomic data is generally high-dimensional, intricate, and unevenly spread in species, breeds, and production systems. Most of the biological effects presented in genomic data are non-linear, situation-specific, and dependent on interactions between the environment and management factors. However, the traditional statistical and machine learning methods are not always useful because they do not always help to address these complexities, especially when data are sparse, skewed, or incomplete. Such restrictions limit the application of genomic knowledge to actionable information in smart agriculture systems and the need to liberalize and express computational approaches (Lourenço et al., 2024; Zhu et al., 2025).

Generative AI has become a strong paradigm in recent years, which can help deal with most of these issues. Generative models are not used to predict particular outcomes, unlike traditional predictive models, which are more concerned with estimating them. The ability enables generative AI to recreate novel, biologically realistic representations and reason about unforeseen circumstances, as well as methodically model uncertainty. This is a conceptual revolution with far-reaching consequences in life sciences, where biological systems are stochastic in nature, hierarchical, and determined by complex interactions (Cao et al., 2025).

Treating genetic variation, regulatory processes, and genotype-phenotype interactions as probabilistic rather than hardwired allows generative AI to offer novel approaches to working with genetic data in animal genomics, in both analysis and design (Pérez-Enciso et al., 2025). With the ability to learn these processes, generative models will be able to augment limited datasets, simulate genetic diversity, and encourage the analysis of external datasets that are exploratory. This is especially applicable in livestock systems, with data of diverse availability across species and populations, and in which there are ethical, economic, and logistical limitations to the intervention of experimental methods. The combination of genomic information with phenotypic, environmental, and management data is also achievable through generative methods, contributing to a more comprehensive view of biological systems through smart agriculture (Liu et al., 2025).

**Fig. 1:**
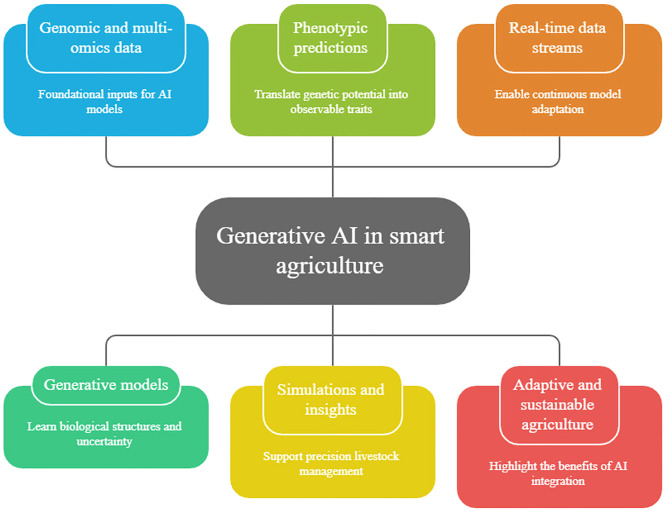
Conceptual framework linking generative AI, animal genomics, and smart agriculture.

The combination of generative AI, animal genomics, and smart agriculture opens up the opportunity to be more anticipatory and system-oriented in the process of livestock management. With the connection between genetic possibilities and the evolving environment and operation, generative models can assist in scenario-based reasoning, adaptive decision-making, and long-term sustainability planning. Simultaneously, there are significant scientific, technical, and social concerns in the use of generative AI in this field. Biological validity, interpretability, fairness, and responsible use should be ensured, especially so because of the multi-generational implications of genomics-informed choices. To overcome these obstacles, strong validation frameworks are needed that include statistical analysis of model performance, biological validation of generated or predicted genomic features, functional assessment through in silico or experimental methods, transparent model design, and adherence to ethical and regulatory principles (Kazembe & Mkandawire, 2024; Shaikh et al., 2025). Fig. 1 illustrates a structured conceptual framework connecting generative AI, animal genomics, and smart agricultural systems via a multi-layered data and modelling pipeline. Genomic, phenotypic, and environmental data streams are integrated into generative modelling architectures where latent representations are learnt to capture complex biological dependencies and variability. The framework emphasizes essential computational components such as data pre-processing, feature encoding, generative modelling, and the generation of synthetic data or sequences. These outputs are then linked to applications such as genotype–phenotype prediction, biological design, and decision support systems in precision livestock farming. Feedback mechanisms are incorporated to iteratively refine model performance using newly generated and observed data, thus enabling adaptive, data-driven agricultural management.

Key concepts and definitions in generative AI and smart animal genomics are presented in Table 1. In addition, the evolution of AI paradigms in animal genomics and smart agriculture is shown in Table 2. Contemporary AI is based on statistical techniques that predate it. These techniques formed the foundation for computational approaches in animal breeding and genomics. The development of Best Linear Unbiased Prediction (BLUP), for example, established a foundation for quantitative genetic evaluation by providing a rigorous framework for estimating breeding values under structured population conditions. The formalization of genomic selection, which allowed for the incorporation of dense molecular marker information into predictive models, signaled a crucial shift toward data-driven breeding techniques and altered this paradigm (Ghavi Hossein-Zadeh, 2024; Boktar, 2025). These developments can be seen as crucial forerunners to modern AI-based approaches because they introduced the concepts of high-dimensional inference, model-based prediction, and integration of genomic information that underpin current machine learning and generative frameworks (Hickey et al., 2017; Johnsson, 2023).

**Table 1:** Key concepts and definitions in generative AI and smart animal genomics.

Concept	Definition in the generative AI context	Definition in the animal genomics context	Relevance to smart agriculture
Generative AI	A class of AI methods designed to learn underlying data distributions and generate novel, realistic data instances rather than solely making predictions or classifications	A computational paradigm applied to model, simulate, or generate biologically plausible genomic, molecular, or phenotypic representations	Enables proactive and adaptive agricultural systems by supporting simulation-driven decision-making, biological design, and scenario exploration
Animal genomics	A domain providing high-dimensional biological data that can be modeled, embedded, and synthesized by generative algorithms	The study of genome structure, variation, and function in domesticated and farmed animal species	Forms the biological foundation for data-driven breeding, health management, and sustainability-oriented livestock systems
Smart agriculture	A cyber-physical system where AI models integrate data streams, automate reasoning, and optimize outcomes under uncertainty	An agricultural paradigm that leverages genomic, phenotypic, and environmental data for precision management	Serves as the operational framework in which generative AI and genomics are deployed for intelligent, adaptive farming
Genotype-phenotype relationship	A complex, probabilistic mapping learned by generative models, capturing non-linear and uncertain biological effects	The biological relationship between genetic variation and observable traits is influenced by environment and management	Central to predicting performance, resilience, and welfare outcomes in livestock production systems
Synthetic genomic data	Artificially generated data instances that statistically resemble real genomic data, while not directly copying original samples	Simulated genomes or variants are used to augment datasets, explore diversity, or protect sensitive genetic information	Supports data augmentation, privacy preservation, and robust model training in data-limited agricultural settings
Latent representation	A compressed, abstract encoding learned by generative models that captures the underlying structure in complex data	An implicit representation of genetic, regulatory, or phenotypic states inferred from high-dimensional biological data	Facilitates integration across genomic, phenotypic, and environmental layers in smart agriculture pipelines
Multi-omics integration	A generative modeling approach that jointly learns from multiple heterogeneous data modalities	The combined analysis of genomic, transcriptomic, epigenomic, and related biological layers	Enables holistic biological understanding and improved trait prediction under real-world farming conditions
Precision livestock farming	An AI-enabled operational domain where real-time data are modeled and interpreted for automated decision support	A management approach using detailed animal-level data to optimize health, productivity, and welfare	Acts as the execution layer where generative genomics-informed insights are translated into management actions
Digital twins	A generative, continuously updated virtual model capable of simulating future biological and management states	A computational abstraction representing the genomic and phenotypic dynamics of an individual animal	Supports predictive management, risk assessment, and sustainability planning in smart agriculture systems
Model generalization	The ability of a generative model to produce valid outputs beyond its training distribution	The biological relevance of predictions across breeds, environments, and production systems	Determines the scalability and reliability of AI-driven genomic tools in diverse agricultural contexts
Bias in AI models	Systematic distortions learned from non-representative or historically constrained data	Overrepresentation of specific breeds, traits, or production systems in genomic datasets	Influences fairness, genetic diversity, and long-term sustainability of smart agriculture solutions
Interpretability	The degree to which the internal logic and outputs of generative models can be understood by humans	The ability to relate model behavior to known biological mechanisms or genomic features	Essential for trust, validation, and responsible deployment in agriculture
Responsible AI	A governance-oriented approach emphasizing transparency, accountability, robustness, and ethical alignment	The application of AI that respects animal welfare, genetic diversity, and societal values	Ensures that generative AI contributes positively to sustainable and socially acceptable agriculture
Sustainability	An optimization objective incorporating long-term system stability rather than short-term performance	A balance among productivity, welfare, environmental impact, and genetic resilience	Serves as the overarching goal guiding the integration of generative AI and genomics in agriculture

**Table 2:** Evolution of AI paradigms in animal genomics and smart agriculture.

AI paradigm	Core conceptual orientation	Primary function in animal genomics	Contribution to smart agriculture
Rule-based and expert systems	• Explicit knowledge encoded as rules • Deterministic reasoning	• Applies expert-defined genotype-trait rules • Supports basic genetic decision logic	• Enables early decision support • Limited adaptability to dynamic systems
Classical machine learning	• Statistical learning from labeled data • Feature engineering dependence	• Marker–trait association and classification • Population-level trait prediction	• Improves automation and consistency • Still largely retrospective and static
Deep learning	• Hierarchical representation learning • Non-linear high-dimensional modeling	• Learns complex genotype-phenotype mappings • Extracts features directly from genomic inputs	• Improves predictive performance • Increases compute needs and reduces transparency
Discriminative AI models	• Learns conditional input-output mappings • Prediction-focused objectives	• Predicts breeding values and risks • Supports trait and disease susceptibility estimation	• Enables precision interventions • Limited scenario simulation and exploration
Generative AI	• Learns full data distributions • Supports synthesis and uncertainty modeling	• Generates synthetic genomes/variants • Models regulatory and phenotypic diversity	• Enables scenario-based planning • Supports individualized adaptive management
Foundation models for biology	• Large-scale pretraining • Transferable general representations	• Produces reusable genomic embeddings • Supports cross-species sequence modeling	• Reduces data barriers • Scales AI tools across diverse systems
Multimodal AI systems	• Joint modeling across modalities • Shared latent representations	• Integrates genomics with phenotypes and environment • Learns context-dependent biology	• Enables whole-system intelligence • Improves coherence of management decisions
Federated and privacy-preserving AI	• Decentralized learning without raw data sharing • Data sovereignty emphasis	• Trains models across distributed genomic repositories • Protects proprietary breeding data	• Enables trusted collaboration • Expands participation across stakeholders
AI-driven digital twins	• Dynamic simulation with continuous updating • Data-driven forecasting	• Maintains virtual genomic-phenotypic states • Simulates longitudinal biological trajectories	• Supports predictive management • Enables resilience and sustainability planning
Responsible and regulated AI	• Ethical/legal/social alignment • Accountability and transparency	• Governs AI-assisted genomic decisions • Enables oversight of breeding and intervention tools	• Builds societal trust • Aligns innovation with welfare and sustainability

In recent years, research at the intersection of AI and genomics has expanded rapidly. However, existing reviews have predominantly focused on discriminative modeling paradigms that emphasize prediction, classification, and association mapping (Çelik, 2024). In contrast, the emerging paradigm of generative AI introduces fundamentally different capabilities centered on data synthesis, probabilistic simulation, and exploration of unobserved biological variation. These capabilities remain underrepresented in current surveys of agricultural and genomic AI. Furthermore, the field of livestock genomics is distinct from human and model organism genomics due to unique biological and operational constraints, including long generation intervals, populations shaped by selective breeding, and strong genotype-environment interactions that complicate data availability and generalization (Jonas & de Koning, 2015). These characteristics necessitate a synthesis dedicated to contextualizing generative modeling within the specific realities of animal production systems. Concurrently, the field is experiencing a significant surge in research activity, fueled by advancements in foundation models, multi-omics integration, and digital agriculture technologies. This has resulted in an expanding body of literature that remains largely unconsolidated. The convergence of methodological innovation and domain-specific complexity underscores the timeliness of this review, which aims to bridge a critical gap by providing an integrative, comprehensive perspective on generative AI in animal genomics for smart agriculture. This review primarily focuses on major livestock species, such as cattle, poultry, and swine, which are the most extensively studied in the context of genomic selection and precision agriculture. Insights from other production systems, such as small ruminants and aquaculture species, are also considered where relevant to highlight broader methodological applicability. This scope reflects the current availability of genomic and phenotypic data, as well as the maturity of computational applications, across different livestock sectors.

This review aimed to present a comprehensive and critical review of the role of generative AI in animal genomics in the context of smart agriculture, in general. The review discusses principles of generative modeling and biological data representation, an overview of the new applications in genomic analysis, breeding, regulation, and system integration, and an assessment of the major challenges of data, computation, validation, and governance. Placing generative AI into the context of operationalization and sustainability objectives of livestock production systems, the review aims to make it clear why generative AI is transformative, as well as what circumstances need to be established to allow responsible integration. It is based on this combined view that the goal of the review is to inform researchers, practitioners, and policymakers on the potential contributions that generative AI will make to next-generation smart agriculture by integrating the innovations of computational perspective with the biological understanding and future agricultural goals. This review treats ethical, legal, and societal considerations as central and cross-cutting themes, reflecting their growing importance in the development and deployment of generative AI in animal genomics.

This review discusses recent advances in generative AI. It is important to note that many of these developments originate from broader domains, such as human genomics and computational biology. The application of these developments to livestock systems varies in maturity, ranging from early conceptual exploration to limited empirical validation. Accordingly, this review distinguishes between established applications and emerging approaches that require further validation in the context of animal genomics.

## 2 Methodological approach and literature search strategy

This review takes a structured narrative approach to synthesize the latest knowledge at the intersection of generative AI, animal genomics, and smart agriculture. While not a systematic review or meta-analysis, the study employs a transparent, reproducible process for identifying literature to ensure comprehensive coverage and minimize selection bias. The methodological framework is guided by the principles of rigor, relevance, and interdisciplinarity to reflect the subject's rapidly evolving and cross-domain nature.

The literature search included peer-reviewed articles published since January 2000, prioritizing those published in 2020 and later. The search was conducted using multiple widely recognized academic and preprint databases to capture peer-reviewed and emerging research. Major indexing platforms, including PubMed, Scopus, and Web of Science, were consulted to identify established studies in genomics, animal science, and computational biology. In parallel, preprint repositories, such as Arxiv and BioRxiv, were included to ensure timely coverage of recent developments in generative AI and genomic modeling. These areas are subject to rapid innovation. The search strategy incorporated combinations of keywords related to generative AI, genomic analysis, livestock systems, and smart agriculture. These terms were iteratively refined to balance sensitivity and specificity, enabling the identification of literature spanning foundational methodological developments, as well as applied research in animal genomics. The temporal scope prioritized recent contributions to reflect the field's current state, while also incorporating earlier foundational works to provide historical and conceptual context.

Inclusion criteria were established to ensure that the selected studies were directly relevant to the review's thematic focus. Articles addressing generative modeling approaches, AI applications in genomics, or the integration of genomic data in agricultural or livestock systems were included. Both methodological and application-oriented studies were considered if they contributed to the understanding of generative AI's role in biological sequence modeling, genotype–phenotype relationships, and smart farming environments. Studies that linked computational innovation with biological or agricultural applications, particularly interdisciplinary ones, were prioritized because they align with the integrative scope of this review. Recent preprints presenting novel methodological contributions or emerging applications not yet fully represented in the peer-reviewed literature were included to ensure the review captures ongoing research at the forefront.

Exclusion criteria were applied to maintain conceptual clarity and focus. Studies limited to purely discriminative machine learning approaches, unless they provided essential contextual or comparative insights, were excluded. Similarly, articles that focused exclusively on human genomics without transferable methodological relevance to animal systems were not included. Publications lacking sufficient methodological transparency or scientific rigor were also omitted. Regarding preprints, selection was based on relevance, methodological soundness, and alignment with established research directions rather than inclusion based solely on recency. This selective approach was adopted to mitigate potential biases associated with the rapid proliferation of unreviewed studies while capturing emerging trends.

Overall, the literature selection process balanced breadth and depth to ensure comprehensive coverage of key developments while maintaining a coherent, focused narrative. Combining systematic search principles with expert-driven curation enables robust synthesis of the current generative AI landscape in animal genomics and its implications for smart agriculture.

## 2 Foundations of generative AI in genomics

### 2.1 Types of generative models

Generative AI models vary not only in their mathematical models but also in their conceptualization and operationalization of the process of biological data generation. In genomics, where patterns of observed sequences and molecular structures are ultimately determined by the interaction of complex biological mechanisms, including mutation, recombination, selection, and regulation, the ability of a model to mirror such complexities becomes of fundamental importance. All generative models have different assumptions regarding the structure of data and its generation, and this affects their appropriateness to model genetic variation, regulation relationships, and population-scale variability in animal genomes. The need to understand these differences further to choose and adapt general generative models to agricultural genomics settings is crucial (Khan et al., 2025).

Generative adversarial networks are based on implicit density modelling, i.e., they are trained to create samples without directly estimating the probability distributions. This property enables them to give highly realistic outputs, but they also restrict their capability to give estimates of uncertainty or probabilistic interpretations. In genomics, such an absence of directly specified likelihoods makes biological validation harder, in that it is difficult to measure the extent to which generated sequences are indicative of underlying evolutionary constraints. In addition, adversarial training is vulnerable to imbalance in the data, which is typical of the animal genomics field, where certain breeds or populations are often underrepresented. Despite these issues, adversarial models are still powerful because of their expressive power and also because they can model high-dimensional and complex genomic characteristics that are hard to explain by lower-level probabilistic models.

Variational autoencoders have a different philosophy of expressly modeling the probability distributions of latent variables. Their explicit probabilistic form is especially appealing to biological interpretation when latent dimensions can be hypothesized to have important sources of genetic variation. Latent representations of a variational model can be used in animal genomics to facilitate the exploratory analysis of population structure, genetic diversity, and variation with respect to traits. The generalization and smoothness suggested by the regularization of the latent prior can be useful where noisy or incomplete genomic data is involved. Nevertheless, the address of the same regularization can be oppressive of the infrequent or extreme genetic designs, which can be biologically significant in the relationship of development to adaptation or disease resistance. Striking a balance between expressiveness and regularization is one of the key issues in relation to the application of variational models to genomics (Cai, 2024; Sun, 2025; Wu & Jang, 2025).

To better understand recent advances in generative AI, it is important to consider the development of generative models designed specifically for biological sequence data. Unlike earlier approaches, which treated genomic information as abstract numerical features, these models operate directly on nucleotide sequences. They learn the probabilistic structure and contextual dependencies that govern genomic organization. Recent developments in generative modeling have introduced architectures that can capture both local motifs and long-range interactions within DNA sequences. This enables a more accurate representation of regulatory grammar and genomic function. In particular, diffusion- and transformer-based generative frameworks have demonstrated the ability to model sequence distributions at scale. These frameworks support the generation of biologically plausible DNA sequences that adhere to underlying structural and functional constraints (Islam et al., 2025; Shehu, 2025; Zhang et al., 2025).

These sequence-centric generative models set a technical standard for the field by shifting the focus from predictive tasks to the synthesis and exploration of genomic variation. By learning latent representations of sequence structure, these models can generate novel sequences with desired properties, such as regulatory activity or structural stability, while maintaining coherence with learned biological patterns. This capability reflects a broader shift toward foundation models in genomics, where large-scale pretraining on diverse sequence datasets supports transferability across tasks and domains. These models offer a unifying framework that integrates multiple downstream applications, including variant prediction, regulatory modeling, and functional annotation, within a single generative paradigm (Shehu, 2025; Zhu et al., 2024a).

Importantly, the adoption of these foundational sequence models in animal genomics demonstrates the intersection of general-purpose generative AI and domain-specific biological research. Although these models were initially developed in broader, human-centric genomic contexts, their principles can be applied to livestock systems. They can be adapted to account for species-specific genomic architectures, breeding histories, and selection pressures. This convergence allows methodological advances to be transferred across domains and motivates the development of specialized models tailored to the unique characteristics of animal genomics. Furthermore, integrating sequence-based generative models into livestock research creates new opportunities to bridge the gap between genotype, regulatory mechanisms, and phenotypic expression. These approaches model genomic sequences as structured and generative entities, facilitating a more mechanistic understanding of how sequence variation influences biological function. This represents a significant departure from traditional marker-based approaches and aligns with the broader transition toward molecular-level modeling in genomics. Thus, these generative models enhance the methodological landscape of AI in biology and serve as a critical bridge between computational innovation and practical applications in smart agriculture (Li et al., 2024; Shoyombo et al., 2024; Islam et al., 2025).

The paradigm of diffusion models is a fundamental change in the way generative processes are conceptualized, since they are characterized by a time-dependent stochastic process. Their denoising process offers a fine-grained control over the generation process, which enables intermediate representations to be viewed and conditioned on auxiliary information. This attribute is in line with the progressive appearance of structure in biological systems and promotes the pliable incorporation of genomic, epigenomic, and phenotypic cues. The diffusion-based methods can be beneficial in the area of agricultural genomics to model continuous biological variation and complex dependencies without the use of adversarial objectives. However, large-scale genomic data puts a practical strain on their computational needs, and methodological methods to adapt diffusion processes to discrete sequence data, such as DNA, are actively being developed (Le, 2024; Das, 2025).

Autoregressive models make more of the element of causality and conditional dependence by explicitly modeling relationships between each component of a sequence and the previous components. This sequential view corresponds to the linear form of genomic sequences, and attention-related mechanisms can be used to capture long-range interactions, which are important in regulatory activity. The introduction of the transformer architecture was a major inflection point in the development of modern generative models. It fundamentally redefined sequence modeling by enabling the efficient learning of long-range dependencies through attention mechanisms. Since then, this architectural innovation has become the backbone of large-scale sequence models, including those adapted for biological data. There, it facilitates the representation of complex genomic patterns and regulatory relationships. The scalability and flexibility of transformer-based models have contributed to the development of genomic language models and accelerated the adoption of generative AI in the life sciences and animal genomics (Sajun et al., 2024; Vangalapat, 2025).

In animal genomics, autoregressive transformers can learn dependencies that cover extensive regions of the genome, which are associated with the impact of distal elements on gene expression and trait variance. Their high probability-based development is easy to evaluate and estimate uncertainty, which is necessary for scientific credibility. However, their dependency on large volumes of training data may be a limiting factor in terms of species or population where the genomic resource is limited, as it often occurs in livestock agriculture (Duan et al., 2025; Fukaya, 2026).

The innovations of large language models bring these principles of autoregression to scale, both in terms of the model structure and training goals, to such an extent that they form a hierarchy of abstractions on top of sequence data. These types of models can be trained to create representations of evolutionary conservation, functional constraint, and contextual relations across genomic regions when applied to biological sequences. They are especially attractive to smart agriculture, where various genomic tasks need to be combined into cohesive decision-support systems, since their ability to act as general-purpose foundation models is especially attractive. Meanwhile, their size poses significant issues on computational accessibility, energy consumption, and reproducibility. The factors impact the viability and fairness of large generative model deployment in agricultural environments, which have diverse resources and infrastructure types and usage (Benegas et al., 2024).

**Fig. 2:**
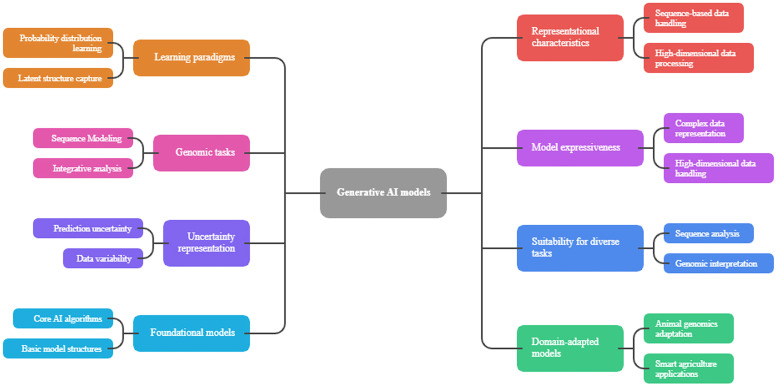
Taxonomy of generative AI models applied to animal genomics.

In any type of generative model, one of the key aspects of genomics is the trade-off between computation abstraction and biological realism. The generative models should be complex enough to represent a real biological variation without overfitting to artifacts and bias in the training data. Generative models used in animal genomics should also be ethically and sustainably sensitive because these disciplines are strongly influenced by selective breeding, population structure, and thus, genetic patterns. There may be long-term effects on agricultural resilience of models that unintentionally increase genetic homogeneity or conceal rare but useful properties (Walters et al., 2025). Fig. 2 presents a taxonomy of generative artificial intelligence (AI) models used in animal genomics. The models are organized according to their underlying computational mechanisms and data modeling strategies. The diagram distinguishes between the following major model classes: generative adversarial networks, variational autoencoders, autoregressive models (such as transformers), and diffusion-based approaches. Each class is annotated with a simplified workflow representation of its internal processes. Adversarial learning, for instance, is depicted through the interaction between generator and discriminator networks, and diffusion models are represented by iterative denoising steps that transform stochastic inputs into structured outputs. The figure also illustrates how these architectures process genomic sequences or multi-omics data to learn probabilistic distributions and generate new biological instances. The comparative positioning of these models reflects their differences in stability, scalability, and suitability for discrete biological data. This provides a structured overview of their roles in genomic applications.

The generative modeling landscape in genomics comprises a variety of architectural paradigms, each with its own unique mechanisms, capabilities, and limitations. Early approaches, such as generative adversarial networks and variational autoencoders, established foundational methodologies. However, more recent developments, including autoregressive transformer-based models and diffusion models, are increasingly shaping the field. In particular, diffusion-based approaches have attracted significant attention due to their improved training stability and ability to model complex biological sequences more effectively than earlier adversarial frameworks. Concurrently, the emergence of hybrid, graph-based, and foundation models reflects a broader shift toward integrative, system-level representations of genomic data. Understanding the comparative strengths and limitations of these model classes is essential for selecting appropriate strategies in animal genomics and evaluating their readiness for practical deployment (dos Santos & Franco, 2025; Hassan et al., 2025). Classes of generative models and their characteristics in genomic applications are listed in Table 3. In addition, a comparison of conventional AI vs. generative AI paradigms in livestock genomics is presented in Table 4.

**Table 3:** Classes of generative models and their characteristics in genomic applications.

Model class	Core mechanism	Strengths in genomic applications	Limitations	Comparative position and current relevance
Generative adversarial networks (GANs)	• Adversarial training between the generator and discriminator • Implicit learning of data distributions	• Generates realistic synthetic genomic data • Useful for data augmentation and class imbalance • Flexible across data modalities	• Training instability and mode collapse • Poor handling of discrete sequence data • Sensitive to hyperparameter tuning	• Early dominant generative approach in genomics • Gradually being superseded by diffusion models • Still useful for specific augmentation tasks
Variational autoencoders (VAEs)	• Probabilistic encoding into latent space • Reconstruction via regularized decoder	• Interpretable latent representations • Stable and efficient training • Useful for dimensionality reduction and feature learning	• Blurred or less precise outputs • Limited ability to capture complex dependencies	• Foundational generative framework • Often integrated with hybrid models • Less competitive for high-fidelity sequence generation
Autoregressive models (transformers and related architectures)	• Sequential token prediction using attention mechanisms • Context-dependent modeling of sequences	• Strong performance in modeling genomic sequences • Captures long-range dependencies and regulatory patterns • Scalable to large genomic datasets	• High computational and data requirements • Sequential generation can accumulate errors • Requires extensive training resources	• Dominant paradigm for genomic language modeling • Highly relevant for sequence-based prediction and generation • Complementary to diffusion models in generative tasks
Diffusion models	• Iterative denoising from noise to structured data • Explicit probabilistic modeling of data distributions	• Improved training stability over GANs • Strong capacity for complex and multimodal distributions • Increasing adaptability to discrete biological sequences	• Computationally intensive • Slower generation process • Still emerging in genomic applications	• Rapidly gaining prominence in sequence generation • Increasingly favored over GANs for stability and robustness • Early-stage but highly promising in animal genomics
Normalizing flow models	• Invertible transformations between data and latent space • Exact likelihood estimation	• Precise density estimation • Bidirectional mapping between latent and data space • Theoretically grounded framework	• Limited scalability to very high-dimensional data • Architectural constraints reduce flexibility	• Niche applications in genomics • Less widely adopted compared to VAEs and transformers
Energy-based models (EBMs)	• Learn energy landscapes representing data distributions • Sampling via optimization or Markov processes	• Flexible modeling of complex distributions • Does not require explicit likelihood functions	• Difficult and computationally expensive training • Challenging sampling procedures	• Primarily theoretical or experimental use • Limited practical adoption in animal genomics
Hybrid and composite models	• Combination of multiple generative frameworks • Integration of deep learning and probabilistic approaches	• Combines the strengths of different architectures • Improves performance in complex tasks • Enables multimodal data integration	• Increased model complexity • Higher computational demands • Difficult optimization and interpretability	• Increasingly important in multi-omics integration • Reflects trend toward system-level modeling
Graph-based generative models	• Operate on graph-structured biological data • Model relationships between genes, proteins, or variants	• Captures relational and network-level genomic information • Suitable for regulatory and interaction modeling	• Computational complexity • Requires high-quality graph representations	• Emerging relevance in systems genomics • Complements sequence-based approaches
Reinforcement learning–guided generative models	• Iterative optimization using reward-based frameworks • Often combined with generative architectures	• Enables goal-directed sequence design • Optimizes functional or biological objectives	• Requires well-defined reward functions • Training instability and computational cost	• Increasingly used in sequence optimization tasks • Still experimental in livestock genomics
Large foundation models (genomic language models)	• Pretrained on large-scale genomic data • Fine-tuned for downstream tasks	• Captures generalizable genomic representations • Enables transfer learning across species and tasks • Supports multimodal integration	• Extremely resource-intensive training • Limited interpretability • Requires large-scale datasets	• Rapidly emerging paradigm • Bridges autoregressive and multimodal modeling • High potential but early-stage in livestock applications

**Table 4:** Comparison of conventional AI vs. generative AI paradigms in livestock genomics.

Comparison dimension	Conventional AI Paradigms	Generative AI Paradigms
Primary modeling objective	• Predicts predefined outputs from given inputs • Focuses on classification or regression tasks	• Learns underlying data-generating distributions • Focuses on data synthesis, simulation, and probabilistic inference
Treatment of genomic data	• Uses observed genomic variants as fixed predictors • Limited exploration beyond existing data	• Models genomic variation as a generative process • Explores plausible but unobserved genetic configurations
Handling of data scarcity	• Performance degrades sharply with limited or imbalanced datasets • Relies heavily on large labeled datasets	• Augments limited data through synthetic generation • Learns structure even under sparse data regimes
Representation of biological complexity	• Often relies on simplified or linearized relationships • Limited capacity to model higher-order interactions	• Captures non-linear, hierarchical, and latent biological structures • Models complex dependencies among genomic features
Uncertainty modeling	• Typically produces point estimates or deterministic predictions • Uncertainty is often implicit or post hoc	• Explicitly represents uncertainty through probabilistic outputs • Generates distributions of possible outcomes
Generalization capability	• Strongly tied to the training data domain • Limited transferability across breeds or environments	• Supports transfer learning and cross-domain inference • Better suited for cross-species and cross-context modeling
Interpretation of genotype-phenotype relationships	• Emphasizes direct associations and feature importance • Often treats effects independently	• Models genotype-phenotype relationships as stochastic processes • Captures emergent and context-dependent effects
Role in genomic selection	• Improves prediction accuracy within known populations • Optimizes selection based on observed data	• Enhances selection through data augmentation and scenario simulation • Supports long-term and risk-aware selection strategies
Ability to generate novel biological hypotheses	• Limited to inference from observed correlations • Hypothesis generation is indirect	• Directly generates novel genomic, regulatory, or phenotypic hypotheses • Enables exploratory and design-oriented research
Integration with smart agriculture systems	• Supports decision-making through static predictions • Limited adaptability to dynamic environments	• Enables adaptive, scenario-based, and anticipatory decision support • Integrates seamlessly with digital twins and real-time systems
Computational characteristics	• Often more computationally efficient and simpler to deploy • Lower training complexity	• Typically more computationally demanding • Requires specialized infrastructure and expertise
Risk profile and failure modes	• Errors usually traceable to model assumptions or data noise • Failures are often localized	• Risks include biologically implausible generation and overconfidence • Failures may propagate across pipelines
Alignment with sustainable agriculture goals	• Optimizes short- to medium-term performance metrics • Limited foresight into long-term impacts	• Supports long-term system resilience and sustainability analysis • Enables evaluation of trade-offs across objectives
Ethical and governance implications	• Ethical concerns mainly related to data use and bias • Governance relatively established	• Raises additional concerns about synthetic biology, design authority, and accountability • Requires evolving regulatory frameworks

### 2.2 Biological sequence representation for AI

Along with the core elements of any AI system used in genomics, biological sequence representation is a fundamental element since how biological information is encoded directly determines what patterns a model is capable of learning, generating, and interpreting. In animal genomics, the biological information is mainly carried by DNA, RNA, and protein sequences, and each is regulated by different structuring principles, functional limitations, and evolutionary forces. The non-trivial nature of task translation into computational representations that retain their biological meaning yet are solvable with generative AI makes translation of these sequences into representations challenging. A suitable representation should strike a balance between simplicity and expressiveness, as well as represent both local sequence characteristics and long-range relationships that have a role in biological activity. Since generative models focus on learning processes that generate data, the sequence representation is a critical factor that determines the biological realism and usefulness of the model in smart agricultural systems (Dong et al., 2025; Pasupuleti, 2025).

On the simplest level, biological sequences consist of discrete symbolic strings of finite alphabets, e.g., in nucleotide sequences in DNA and RNA or amino acids in proteins. Direct symbolic representations are biologically intuitive, but not all machine learning architectures are compatible with direct symbolic representations, and many machine learning architectures are numeric (numerical) in nature. As a result, sequences have to be encoded in numerical form to enable the models to process, compare, and create biological data. Simple encoding schemes offer a direct mapping between symbols and number vectors, which actually retain sequence identities and allow sequence manipulation. These representations, however, do not capture biochemical similarities, evolutionary relationships, or functional context because they represent symbols that are independent entities. This weakness is especially sharp when it comes to generative modeling, where we not only want to see the patterns but also create new sequences that abide by biological laws (Hu et al., 2025; Zhang et al., 2025).

More expressive representations attempt to encode biological sequences in a continuous vector space in which sequence elements can be learned to have meaningful relationships with each other. Embedding methods seek to encode discrete biological symbols or subsequences into a numerical form of representation via which similarities are determined with respect to contextual usage, not by a priori regularity. In genomics, this method enables the models to learn a representation where functionally related or evolutionarily preserved things are in proximity in latent space. These embeddings are particularly useful when using generative AI, where it is possible to interpolate, extrapolate, and sample in biologically meaningful subsets of the representation space. In the case of animal genomics, embedding-based representations can be used to model intricate genetic variation among populations, and are useful for computing tasks that cannot be done with observed sequences alone (Feng et al., 2025; Zhang et al., 2025).

A useful conceptual framework for understanding the evolution of computational approaches in animal genomics is the distinction between matrix- and molecule-based perspectives. Matrix-based approaches originate from classical quantitative genetics and represent genomic information through structured numerical matrices that encode relationships among individuals, markers, or populations. These representations typically emphasize aggregate statistical properties, such as covariance structures and genetic similarity. They have historically been used to estimate breeding values and predict phenotypic outcomes. In this framework, genomic data are treated as high-dimensional yet ultimately abstract numerical inputs. The analytical focus is on optimizing predictive accuracy through statistical inference and model fitting (Johnsson, 2023).

In contrast, molecule-based perspectives conceptualize genomic data as biologically meaningful sequences of ordered nucleotides where functional information is embedded in the arrangement, context, and interaction of sequence elements. This approach emphasizes the intrinsic structure of DNA, including motifs, regulatory regions, and long-range dependencies that govern gene expression and biological processes. Instead of reducing genomic information to summary matrices, molecule-based approaches aim to preserve and model the underlying biological complexity of sequences. This enables a more direct interpretation of how genetic variation influences function. This shift reflects a broader movement toward a mechanistic understanding where computational models are used to predict and elucidate the processes that generate outcomes (Johnsson, 2023; Dai, 2025).

The emergence of generative AI has significantly accelerated the shift toward molecule-based modeling by offering tools that can learn and reproduce the probabilistic structure of biological sequences. Sequence-based generative models, especially those based on transformer and diffusion architectures, work directly with nucleotide-level data and capture local and global dependencies within genomic sequences. These models can then be used to simulate regulatory grammar, epistatic interactions, and context-dependent effects, which are difficult to represent within matrix-based frameworks. Thus, generative AI enables a more nuanced, biologically grounded representation of genomic information, bridging the gap between statistical abstraction and molecular detail (Islam et al., 2025).

Importantly, these two perspectives should be viewed as complementary components of a unified analytical framework, not as mutually exclusive. Matrix-based approaches are still very effective for large-scale population analysis, estimating breeding values, and integrating pedigree and genomic information, especially in applied livestock systems. Meanwhile, molecule-based models provide deeper insight into the functional and mechanistic aspects of genomic variation. These models support applications such as regulatory modeling, sequence design, and variant interpretation. Integrating these perspectives provides a multi-scale understanding of animal genomics, considering both population-level patterns and molecular-level mechanisms (Johnson, 2023; Ren et al., 2024).

In the context of smart agriculture, this conceptual integration is valuable because it aligns predictive modeling, mechanistic insight, and practical decision-making. Generative AI bridges the gap between matrix- and molecule-based paradigms by incorporating sequence-level information into broader analytical workflows that inform breeding, management, and system optimization. The present review frames the evolution of genomic analysis in terms of these complementary perspectives, providing a coherent lens through which diverse computational approaches can be understood and applied to develop intelligent and sustainable livestock systems (Johnson, 2023; Garcia-Oliveira et al., 2026).

The maintenance of positional information is a critical problem in the representation of biological sequences. In contrast to most applications of natural languages, where the order of words can give meaning but is not too strict, the position of elements in biological sequences may have an exact functional value. Regulatory motifs, coding regions, and structural domains not only rely on the sequence composition but also on the relative and absolute location. Generative models should thus have in place mechanisms that enable them to differentiate similar symbols that are used in different contexts. Positional encoding techniques offer a way of combining order and distance information into sequence representations so that models can learn local motifs and long-range interactions. It is of particular importance in animal genomics, where regulatory effects can be large-scale in their impacts and provide outcomes of significant agricultural interest (Yang & Wang, 2025).

In addition to linear sequence representations, biological systems are organized by nature in the form of networks of interactions that span across various scales. Genes are regulated by interacting with each other, proteins are formed by interacting with each other, and genomic regions are structured in three-dimensional chromatin structures. The representation of genomic data using linear sequences only would be risky, as it would easily miss these higher-order associations. Graph-based representations provide an effective alternative representation through encoding biological entities in the form of nodes and their interactions in the form of edges, thus explicitly representing relational structure. Graphs may be used as a regulatory interaction, a pattern of co-expression, or a pattern of functional association in the genomics context, and give generative modeling a richer substrate. In the case of smart agriculture, where predicting complex traits requires the knowledge of the interactions at the system level, graph representations allow models to progress beyond the context of individual sequence elements towards system-level biological systems (Thompson et al., 2025; Yuan et al., 2025).

Generative AI especially uses graph-based representations since they enable the representation of non-Euclidean data structures that model biological reality better. Graph-based generative models can be trained to generate new network topologies or be trained to alter topologies and keep their topological and functional features. Such capabilities are useful in animal genomics to study genetic architectures of complex traits, which are commonly a result of interactions between many genes and regulatory elements. Nevertheless, the graph representations further add to computational complexity and demand the meticulous definition of nodes, edges, and attributes to have biological relevance. Graph structure design then becomes a component of the modeling process and not a technical one (Robben, 2025).

The other valuable aspect of biological sequence representation is the combination of multiple layers of biology. The potential is encoded in DNA sequences, dynamic regulation in RNA, and functional outcomes in proteins, which each offer a perspective of biological systems to be understood as complementary. Representations that maintain these layers independently are at risk of missing important interactions between them, which determine phenotype. Multilevel representations are designed to take into account information on multiple scales of molecules to enable generative models to learn the effects of variation at one scale on others. In the case of animal genomics in smart agriculture, these integrated representations are specifically favored, since the traits in agriculture are often complex cascades between the genotype and the phenotype in the environment. Representations that facilitate this integration have advantages in the generative and analysis of biologically consistent multi-omics patterns, which is the benefit of generative AI (Adam et al., 2025; Baião et al., 2025).

The biological sequence representation should also be able to consider the variability and uncertainty present in genomic data. The error of sequencing, lack of information, and population heterogeneity all bring noise that can confuse generative models when not handled appropriately. Strong representations attempt to put the implicit or explicit uncertainty in representations so that models can tell the difference between signal and noise. In generative models based on probabilistic reasoning, representations may be represented in between latent spaces to represent uncertainty and variability, and have more robust generation and inference. Such robustness is needed in ensuring biologically plausible and agriculturally meaningful generative outputs in scenarios where data quality and coverage may be highly variable across species and production systems, as is the case in animal genomics (Zhang et al., 2025).

In practice, the selection of the sequence representation has a dual impact on the performance, interpretability, and usability of the models used in smart agriculture systems. Representations that are closely related to the biological concepts are easier to interpret by domain experts, which helps to build trust and adoption. On the other hand, high abstract representations can result in high predictive or generative capabilities and blur the biological meaning, limiting their usefulness in practice. A key problem in the design of representations in generative AI in genomics is to find a balance between the power of expression and the ability to interpret them. This balance is especially relevant to situations related to agriculture, where choices based on AI models might have long-run implications on breeding practices, animal welfare, and sustainability (Ranaldi, 2025). Biological sequence representation strategies for generative AI in genomics are shown in Table 5.

**Table 5:** Biological sequence representation strategies for generative AI in genomics.

Representation strategy	Core encoding principle	Information captured	Advantages of generative AI	Limitations and challenges
One-hot encoding	• Discrete binary representation of nucleotides or amino acids • Position-specific encoding	• Exact sequence identity • Local positional information	• Simple and lossless representation • Widely compatible with many generative architectures	• High dimensionality • Does not capture similarity between symbols
k-mer based representation	• Fragmentation of sequences into fixed-length subsequences • Frequency or presence-based encoding	• Local motif composition • Short-range dependencies	• Captures biologically meaningful motifs • Reduces sequence length complexity	• Loses long-range positional context • Sensitive to the choice of k
Numerical and physicochemical encoding	• Mapping symbols to numerical or biochemical property vectors	• Biophysical and chemical characteristics • Functional tendencies	• Introduces biologically informed priors • Enhances functional modeling	• Property selection may be subjective • Limited coverage of regulatory information
Embedding-based representations	• Learned dense vector representations • Context-dependent encoding	• Semantic and contextual relationships • Sequence similarity beyond exact matches	• Compact and expressive • Well-suited for large generative models	• Requires large training datasets • Reduced interpretability of learned features
Positional encoding schemes	• Explicit encoding of sequence position • Relative or absolute position modeling	• Order and distance information • Long-range dependencies	• Enables modeling of regulatory interactions • Essential for transformer-based models	• Adds representational complexity • Design choices affect model behavior
Graph-based representations	• Nodes and edges encode biological relationships • Non-linear sequence structure	• Regulatory interactions • Structural and relational dependencies	• Captures complex genomic architecture • Supports multi-scale modeling	• Computationally intensive • Graph construction may introduce bias
Multi-scale representations	• Simultaneous encoding at multiple resolutions • Hierarchical abstraction	• Local motifs and global context • Cross-scale interactions	• Improves robustness and biological realism • Enhances generative diversity	• Increased model complexity • Challenging optimization
Multi-omics integrated representations	• Joint encoding of genomic and auxiliary omics data • Cross-modal alignment	• Regulatory state • Functional and epigenetic context	• Enables holistic biological modeling • Supports phenotype-aware generation	• Data heterogeneity • Alignment and normalization challenges
Pretrained foundation embeddings	• Large-scale unsupervised pretraining • Transferable representations	• Evolutionary constraints • Conserved functional patterns	• Strong performance in low-data regimes • Cross-species generalization	• High computational cost • Risk of embedding dataset bias

### 2.3 Genomics data in livestock species

The empirical basis of the generative AI models in the smart agriculture domain is genomics data in livestock species, which can be developed, trained, and tested. Livestock genomics is a diverse area that encompasses many different species, production systems, breeding histories, and environments, unlike many other areas where data are relatively homogeneous. Cattle, poultry, swine, sheep, and aquaculture species differ in their biological properties, population structures, and genomic architecture, which affects the nature of accessible data as well as the issues posed by their analysis. The implications of these differences on the use of generative AI are far-reaching, since the models should be able to adapt to the heterogeneous distribution of data, while maintaining biologically meaningful variation to agricultural decision-making (Eckhardt et al., 2025; Zhu et al., 2025).

Genomic data in livestock are influenced by species-specific evolutionary histories and domestication, which have caused distinctive genomic variations in genetic diversity spread and linkage construction. Prolonged artificial selection has led to extreme genetic stratification within and outside breeds, and intensive breeding methods have introduced different levels of allotropism and genetic bottlenecks. Genomic variation can be very extensive but may not be uniformly distributed among populations of species with large effective population sizes and short generation times, such as poultry and certain aquaculture species. Conversely, the species with longer generation times, as well as species whose breeding programs are more centralized, tend to have more pedigree levels as well as more pronounced population stratification. These properties have an impact on the representation of genomic variation in data and the way generative models have to consider the population structure, relatedness, and history of selection (Ghavi Hossein-Zadeh, 2024; Nayak et al., 2024).

In addition to genomic sequences, livestock data ecosystems are now being augmented with massive phenotypic data, which includes production, health, behavioral, and adaptability traits. Phenotypic data are complex in nature, and they represent the interplay between genetic potential and the environment, human activity and management, and stochastic biological events. In smart agriculture, digital monitoring technologies often measure phenotypes with high temporal resolution. This generates large, heterogeneous datasets that complement genomic information. For instance, wearable sensors and automated milking systems capture physiological and productivity traits. Computer vision platforms and depth-imaging systems enable the continuous assessment of animal behavior, body condition, and health status. These technologies also allow for the continuous assessment of animal behavior and health status, including monitoring of feeding and drinking behavior, detection of mortality events, and identification of management-relevant outcomes such as floor eggs in poultry systems (Yang et al., 2025a, 2025b). Nevertheless, the phenotypic data are often messier and context-taxing than the genomic data, which causes even more difficulties in integrating and modeling. Generative AI should thus be in a position to address these variations in scale, reliability, and time structure, in learning joint genotype-phenotype representations (Venegas et al., 2025; Zhu et al., 2025).

Combined multi-omics data also adds value to the genomic map of livestock species. Transcriptomic, epigenomic, proteomic, and metabolomic data provide dynamically changing perspectives of biological processes that mediate the interaction between the genotype and phenotype. Such layers of data record regulatory activity, interactions between molecules, and physiological states that cannot be deduced by using the DNA sequence alone. In livestock systems, multi-omics data are typically recorded under special experimental or production conditions, resulting in high-dimensional datasets but limited in scope. This imbalance offers opportunities as well as challenges to generative AI, which can use multi-omics information to learn richer biological representations, but also has to deal with data-type sparsity and heterogeneity (Wadood et al., 2025; Xing et al., 2025).

Economic, logistic, and ethical factors are very powerful determinants of data availability in livestock genomics. Commercially attractive genomic datasets of large scale are more easily obtained for species that have commercial value, as well as for production characteristics, whereas data on minor breeds, large-scale systems, or novel aquaculture species are often scarce. This skewed data underrepresentation results in biased information, which can be amplified by generative models without being taken into account. The generative AI capacity to generalize beyond well-characterized populations is critical to smart agriculture, in which the resilience and sustainability of genetic diversity and adaptability are determined by the ability to generalize. To deal with these disparities, it is necessary to pay special attention to training data curation and creating models that can be trained on small or imbalanced datasets without a reduction in biological validity (Robben, 2025; Shaikh et al., 2025).

Over the last ten years, concerted efforts by international entities have greatly widened the availability and standardization of information about livestock genomes. Massive consortia and reference programs have also created new common resources of high-quality genomic data in many species and populations. These efforts have helped in the enhancement of genome assemblies, or useful annotations, and comparative datasets that enable essential research as well as applied breeding efforts. These resources are especially useful to generative AI, since they contain a wide variety of well-characterized training data usable to train models to learn a strong representation of genomic structure and function. Meanwhile, variations in the data format, annotation guidelines, and access control are also issues, and they make the incorporation of multi-source datasets difficult (Xu et al., 2024; Zhu et al., 2025).

The nature of livestock genomic information has also been an indication of the practicality of agricultural systems. Genotyping platforms, sequencing depth, and sampling plans are not always optimized on a cost-efficient basis, as opposed to comprehensive coverage, which has led to the production of datasets that may have missed information or have not been resolved evenly across the genome. Furthermore, data are often recorded over a long period of time, where breeding objectives, management systems, and environmental variables might evolve. These temporal dynamics add even more complexity to consider, which generative AI must face when modeling genomic variation and the phenotypic outcomes that result from it. The models that do not take into account such context can produce some statistically plausible outputs that are biologically or agriculturally incorrect (Thallman et al., 2025).

An especially demonstrative example of the opportunity and difficulty of livestock genomics is the aquaculture species. Extensive growth in aquaculture has kept pace with more rapid growth in genomic resources of many species, resulting in highly incoherent datasets of varying quality. Simultaneously, the environmental sensitivity and biological diversity of aquatic life pose a high motivation towards genomics-based optimization. Generative AI is potentially beneficial in this regard as it would allow the transfer of knowledge across species and populations; the key is that representations must be able to accommodate a wide biological diversity and limited data coverage. This highlights the fact that there is a wider requirement for adaptable generative structures that are resilient to the peculiarities of various livestock systems (Dhillon et al., 2025; Ibrahim et al., 2025).

The data obtained in the field of livestock genomics has value not merely in the biological content, but also in the ability to be incorporated into decision-support systems, as far as smart agriculture is concerned. The genomic data are becoming increasingly interconnected with management records, environmental indicators, and economic metrics, which constitute intricate data ecosystems that indicate the complex nature of agricultural production. These diverse data streams may be combined by generative AI to create consistent models of system behavior, but doing so requires a thorough understanding of the biases, limitations, and structure of the livestock genomics data. In the absence of this knowledge, generative outputs can become non-congruent with viable agricultural agendas or ethics (Tedeschi et al., 2025).

The evolution of genomic research in animal breeding and agriculture can be understood through a broader bibliometric and intellectual trajectory reflecting the field's progressive expansion and transformation. Early developments in quantitative genetics established a theoretical foundation centered on the statistical modeling of heritable traits. The subsequent integration of molecular markers marked a shift toward genome-informed breeding strategies. As sequencing technologies advanced and high-throughput data generation became more accessible, the volume, diversity, and complexity of genomic datasets increased substantially. This necessitated the adoption of more sophisticated computational approaches. This transition was accompanied by a growing convergence between genomics and data science as machine learning techniques were introduced to address challenges associated with high-dimensional data, nonlinear relationships, and multifactorial trait architectures (Çelik, 2024; Shoyombo et al., 2024).

In recent years, this trajectory has accelerated with the emergence of AI as a central analytical paradigm in genomic research. The growing abundance of multi-omics data, alongside advancements in computational infrastructure and algorithmic innovation, has led to a rapidly growing body of literature at the intersection of genomics and AI. Within this evolving landscape, generative AI is a significant development because it introduces capabilities that extend beyond traditional predictive modeling to include the synthesis, simulation, and exploration of biological data. The growing prominence of these approaches is evident in the increasing number of studies focusing on generative models, sequence-based learning, and integrative frameworks linking genomic variation to phenotypic and environmental contexts (Ahmed et al., 2024; Islam et al., 2025).

This bibliometric expansion reflects not only increased research activity but also a conceptual shift in how genomic data are interpreted and utilized. The field is transitioning from descriptive and predictive paradigms to more integrative and generative frameworks that emphasize system-level comprehension and the capacity to model intricate biological processes. This shift is particularly relevant in animal genomics due to the need to address challenges associated with heterogeneous data sources, diverse production environments, and long-term breeding objectives. The convergence of these factors has created a critical juncture in which knowledge is expanding rapidly yet becoming increasingly fragmented across disciplines (Adebayo et al., 2024).

There is an increasing need for comprehensive syntheses that integrate developments in statistical genetics, machine learning, and generative modeling into a coherent framework. Situating generative AI within this broader bibliometric and intellectual context, the present review addresses the field's increasing complexity and interdisciplinarity. The review aims to consolidate emerging insights, clarify methodological trends, and provide a structured perspective to guide future research and applications in smart agricultural systems. In doing so, the review addresses a timely gap in the literature where rapid innovation has outpaced the development of integrative, domain-specific reviews. Characteristics of genomic and multi-omics data across major livestock species are presented in Table 6.

**Table 6:** Characteristics of genomic and multi-omics data across major livestock species.

Livestock species	Genomic data characteristics	Phenotypic and production data	Multi-omics data landscape	Implications for generative AI applications
Cattle	• Large and well-annotated reference genomes • Extensive population-scale variant datasets	• Detailed records on growth, reproduction, health, and productivity • Longitudinal and management-linked traits	• Increasing availability of transcriptomic and epigenomic data • Moderate integration of metabolomics	• Supports training of large and complex generative models • Enables data augmentation and cross-breed generalization
Poultry	• Compact genomes with high recombination rates • Strong selection signatures	• High-throughput performance and efficiency traits • Rapid lifecycle phenotyping	• Transcriptomic data relatively abundant • Limited epigenomic and metabolomic coverage	• Well-suited for fast iterative generative modeling • Challenges in modeling long-term regulatory effects
Swine	• High genetic diversity across breeds • Well-characterized structural variation	• Comprehensive production, health, and carcass traits • Variable environmental sensitivity	• Growing transcriptomic and proteomic resources • Emerging microbiome data	• Enables modeling of complex genotype-environment interactions • Supports multimodal generative approaches
Sheep and goats	• Moderate genome annotation quality • Diverse local and adaptive populations	• Traits related to adaptation, reproduction, and resilience • Often sparse or heterogeneous records	• Limited but expanding transcriptomic datasets • Sparse epigenomic information	• Requires transfer learning and data augmentation • Suitable for conservation-focused generative modeling
Aquaculture species	• Highly variable genome sizes and structures • Frequent polyploidy in some species	• Traits strongly influenced by environment and husbandry • High phenotypic plasticity	• Uneven availability of transcriptomics and metabolomics • Limited integrative datasets	• Presents challenges for model generalization • Benefits from multimodal and simulation-based generative AI
Minor and indigenous breeds	• Sparse and fragmented genomic data • Underrepresented genetic diversity	• Limited standardized phenotypic records • Strong local adaptation traits	• Minimal multi-omics coverage • Data is often context-specific	• Strong need for synthetic data generation • Ideal use case for transfer learning and privacy-preserving models

## 3 Applications of generative AI in animal genomics

Generative AI has quickly become a powerful and multifaceted collection of solutions to tackle well-known problems in the field of animal genomics, especially in the case of intelligent farming. Contrary to older methods of analysis, which are mainly concerned with prediction or classification, generative models are designed to learn the underlying distributions and latent structures that describe bio-data. Such a change allows one not merely to make better inferences on available data, but also to create new information that is biologically plausible and that can increase the analytical range, aid the formation of hypotheses, and augment system-level decision-making. In the field of animal genomics, where data is often high-dimensional, non-homogeneous, and unevenly distributed across species and production systems, generative AI can help bridge the gap between data and its practical application in agriculture by offering a more flexible framework. In this context, a defining characteristic of generative AI is its ability to generate new data instances, sequences, or representations. This distinguishes it from traditional predictive models, which are limited to making inferences based on existing observations (Pérez-Enciso et al., 2025; Zhu et al., 2025).

### 3.1 Data augmentation and synthetic genomic data

Some of the best-known and most effective uses of generative AI in animal genomics include data augmentation and synthetic genomic data generation. Genomic studies of livestock are often characterized by limitations, including small sample sizes, high costs of data production, and biased samples of breeds, population, or production systems. Such constraints decrease the statistical strength and external validity of downstream models to be applied to genomic prediction, disease risk measurement, and optimization of disease management. Generative AI has the potential to address these challenges. It can learn the joint distributions of genomic features and generate synthetic data that approximates the structure, variability, and dependencies observed in real genomes. However, the reliability and biological validity of such synthetic data are still being investigated, especially in the field of livestock genomics. The purpose of this is to achieve the effective expansion of effective sample sizes using absolutely no corresponding increase in experimental or field data collection, which in turn contributes to more powerful analytical pipelines in intelligent agriculture systems (Miller et al., 2024; Pérez-Enciso et al., 2025).

The usefulness of synthetic genomic data is that generative models can infer a great deal of complex correlation between genetic variants, including that of a population structure, a history of selection, and relationships of genetic links. Instead of merely adding random noise or merely predicting the observations made so far, generative models strive to (biologically) reproduce patterns that have been observed in real genetic variation. Synthetic genomes would be effective synthetic proxies of empirical data, which could be used in training and evaluation processes when properly implemented. This is an improvement of predictive models in smart agriculture that are more capable of being used in a wide variety of contexts and under uncertainty conditions that are needed in resilient and adaptive livestock systems (Szatkownik et al., 2024; Min et al., 2025).

Generative AI data augmentation is of interest to data that is underrepresented and rare, as this information is often pushed to the periphery in traditional genomic data analysis because of the lack of relevant data. Generative models can be used to reduce biases due to overreliance on well-characterized populations by increasing the representation in training datasets. This is in line with the overall objectives of smart agriculture, which are inclusivity, flexibility, and system-wide efficiency as opposed to the narrow returns on performance in densely researched environments. It is possible to increasingly use synthetic genomic data to help make modeling outcomes more equitable and to facilitate innovation in a wider variety of livestock systems (Gogineni & Mupparaju, 2025).

Another benefit of synthetic genomic data is the possibility of sharing data without violating privacy. Genomic data of livestock has a tendency to have commercial significance and be under proprietary rights, which restricts cooperative effort and slows innovation. Generative AI provides an opportunity to generate synthetic data sets that have important statistical characteristics of the original data, and the probability of sensitive information is lower. These datasets will be able to facilitate shared research and model construction, in the presence of proprietary genetic resources, and assist in the creation of more open and interoperable data ecologies in smart agriculture (van Breugel et al., 2024).

Although these are the advantages, synthetic genomic data usage prompts critical methodological and ethical issues. Generative models are necessarily informed by the training data, and empirical biases or gaps can be reproduced or increased by synthetic last products. Otherwise, it will result in the creation of falsified images of genetic diversity or reinforce the status quo in breeding preferences. In animal genomics, where choices affect the genetic make-up of the population over the long term, and where the welfare of animals relies on them, it is necessary to ensure that synthetic data generation is sustainable, as opposed to maximizing profits in the short-term. Responsible data augmentation practices, therefore, require rigorous evaluation, transparency, and governance (Susser et al., 2024; van Breugel et al., 2024). Unlike traditional predictive models, which rely solely on existing datasets, generative approaches allow for the creation of synthetic genomic data. This expands the available data space and addresses limitations related to data scarcity and imbalance. Applications of generative AI for genomic data augmentation and synthetic genome generation are shown in Table 7.

**Table 7:** Applications of generative AI for genomic data augmentation and synthetic genome generation.

Application area	Objective of data augmentation	Generative AI role	Key benefits	Risks and considerations
Augmenting limited genomic datasets	• Increase effective sample size • Reduce sparsity in underrepresented populations	• Learns genomic data distributions • Generates synthetic yet realistic sequences	• Improves robustness of downstream models • Enhances generalization across populations	• Risk of overfitting to training biases • Potential amplification of existing data imbalance
Balancing population structure	• Mitigate skewed breed or line representation • Improve model fairness	• Generates samples reflecting diverse genetic backgrounds • Smooths uneven population distributions	• Reduces population-specific bias • Supports equitable genomic modeling	• May obscure true population stratification • Requires careful validation
Privacy-preserving genomic sharing	• Enable collaboration without exposing sensitive data • Protect proprietary genetic information	• Produces non-identifiable synthetic genomes • Preserves statistical properties of original data	• Facilitates data sharing and federated research • Lowers legal and ethical barriers	• Risk of information leakage if models memorize data • Privacy guarantees must be evaluated
Enhancing rare variant representation	• Improve learning of low-frequency genetic variants • Support rare-trait modeling	• Simulates plausible rare variants within learned distributions • Expands variant diversity	• Strengthens detection of rare genetic effects • Improves predictive coverage	• Synthetic rarity may not reflect biological reality • Validation remains challenging
Supporting robust model training	• Reduce overfitting • Improve stability of AI models	• Supplies diverse training examples • Regularizes learning through variability	• Enhances performance under noisy conditions • Improves reproducibility	• Poor-quality synthetic data can degrade models • Requires quality control mechanisms
Cross-species and cross-breed transfer	• Compensate for missing data in understudied species or breeds • Enable knowledge transfer	• Generates aligned synthetic genomes guided by related species data • Bridges data gaps	• Accelerates research in data-poor systems • Supports inclusive smart agriculture	• Risk of biological mismatch • Requires evolutionary and contextual constraints
Scenario simulation and stress testing	• Explore hypothetical genetic configurations • Assess model sensitivity	• Generates controlled synthetic variations • Enables in silico experimentation	• Supports risk-aware decision-making • Improves system robustness	• Simulations may oversimplify complex biology • Interpretation must be cautious
Benchmarking and method validation	• Provide controlled datasets for testing algorithms • Enable reproducible evaluation	• Produces standardized synthetic benchmarks • Enables systematic comparison	• Improves transparency in method development • Supports fair model assessment	• Synthetic benchmarks may not capture full biological complexity

### 3.2 Predicting novel genetic variants

Unlike predictive models, which estimate the effects of known variants, generative models propose previously unobserved variants by exploring the underlying distribution of genomic sequences. Therefore, predicting new genetic variants is a prospective use of generative AI that goes beyond the analysis of genomes to the data it observes. The methods of variant discovery that have been traditionally used are limited by nature by the range and size of sequenced populations, making it possible to infer variation that is already found in available datasets. Generative AI is a paradigm that complements traditional methods as it describes the distributions and constraints defining genomic variation, which aspect can be explored to generate plausible variants that have so far not been empirically seen. This generative view is in support of a more active attitude towards the knowledge of genetic possibilities and adaptation in livestock systems (Liu et al., 2025).

Generative models can learn the dependencies and contextual interactions in genomic sequences and make an inference of which sequence mutations are likely to be tolerated or functional in a particular genomic context. This ability allows one to explore variant space systematically and rank possible variants that may be of interest to investigate further. Generative predictions have been useful as a computational filter in areas of animal genomics where experimental validation is expensive and time-consuming, such as informing research and breeding priorities. In the context of smart agriculture, this foresight can be used to make long-term decisions and take calculated risks (Merchant et al., 2025).

The generative AI has been applied especially in predicting regulatory variants, the role of non-coding regions in the regulation of genomic expression, and phenotypic outcomes is central. Regulatory sequences are defined by complicated structures and context-specific actions, which pose difficulties for the standard methods of analysis. The generative sequence models are also ideally applicable when it comes to describing these patterns because they can train distributed representations of regulatory grammar and long-range interactions. To fine-tune the optimization of traits in livestock systems, generative AI can generate plausible variations of regulations. This helps conceptualize how subtle changes in sequence alter biological functionality (Eckhardt et al., 2025; Wang et al., 2025).

Generative variants prediction is also relevant towards enhancing sophisticated traits and illness resilience. Agronomically significant traits are often regulated by small interacting effects that are represented by many variants. The discovery of variant configurations that can be in line with the desired outcomes can be better achieved through generative AI than through conventional methods, since the former can explore this combinatorial space better. The capabilities allow strategies to be designed with foresight of the future challenges in the context of smart agriculture, where resilience and adaptability are becoming key factors (Ajith, 2024).

Simultaneously, novel variant prediction by generative means also brings biological validity and interpretability issues, as well as ethical governance. Not every statistically plausible variant is biologically attainable and even desirable, and the output of a generation method should always be viewed as a hypothesis and not a prescription. It is necessary to combine the generative predictions with biological knowledge, empirical validation, and system-level considerations to prevent unintended consequences. Maintaining transparency in the process of variant generation and prioritization is another way of ensuring responsible application in the agricultural context (Frank et al., 2025). Generative AI approaches for predicting novel genetic and regulatory variants are presented in Table 8.

**Table 8:** Generative AI approaches for predicting novel genetic and regulatory variants.

Variant prediction task	Biological focus	Generative AI strategy	Potential benefits	Key challenges and risks
De novo genetic variant discovery	• Single-nucleotide and small structural variation • Sequence-level novelty	• Learns genome-wide variant distributions • Generates statistically plausible unseen variants	• Expands searchable genetic space • Supports discovery beyond observed datasets	• Risk of generating biologically implausible variants • Requires stringent biological validation
Allele design for trait improvement	• Functional alleles influencing complex traits • Polygenic architectures	• Generates candidate allelic configurations conditioned on desired outcomes • Explores combinatorial variant space	• Enables hypothesis generation for breeding strategies • Supports targeted genomic exploration	• Functional effects may be context-dependent • Ethical and regulatory constraints
Regulatory variant prediction	• Variants affecting promoters, enhancers, and regulatory elements • Non-coding genome	• Models sequence-to-regulation relationships • Generates variants with predicted regulatory impact	• Improves understanding of gene regulation • Enhances prediction of expression-level variation	• Regulatory mechanisms are cell- and context-specific • Validation is complex
Variant effect simulation	• Functional consequences of sequence changes • Genotype-phenotype mapping	• Simulates phenotypic outcomes of generated variants • Captures uncertainty in effect sizes	• Supports risk-aware decision-making • Enables in silico screening	• Simulations may oversimplify biological pathways • Model bias can distort effects
Rare and low-frequency variant modeling	• Variants with limited observational data • Rare trait associations	• Augments representation of rare variants within learned distributions • Enhances detection sensitivity	• Improves learning in sparse data regimes • Supports rare trait analysis	• Synthetic rarity may not reflect evolutionary constraints • High false-positive risk
Cross-population variant transfer	• Variants shared or adapted across breeds or species • Evolutionary conservation	• Generates variants guided by related populations • Transfers learned patterns across genomic contexts	• Enables prediction in understudied populations • Supports inclusive genomic research	• Population-specific effects may be lost • Requires careful evolutionary grounding
Variant prioritization and ranking	• Functional relevance and downstream impact • Regulatory importance	• Generates and scores candidate variants jointly • Learns implicit importance patterns	• Accelerates variant filtering pipelines • Reduces experimental burden	• Rankings depend on training data bias • Interpretability remains limited
Exploration of hypothetical regulatory landscapes	• Unobserved regulatory configurations • Gene expression control	• Generates alternative regulatory sequences under constraints • Enables scenario exploration	• Advances understanding of regulatory flexibility • Supports system-level modeling	• Hypothetical outputs may lack biological feasibility • Requires conservative interpretation

### 3.3 Protein and gene sequence design

Protein and gene sequence design is one of the most ambitious applications of generative AI to animal genomics, where computational analysis is designed as the interpretation into biological design. This generative capability goes beyond prediction, enabling the design of entirely novel sequences with desired properties rather than merely evaluating existing ones. Conventional methods of designing sequences are based on empirical screening or trial and error of known sequences, which constrains the search of the high-combination space of possible biological arrangements. Generative AI provides a radically novel methodology based on learning the statistical and functional constraints governing sequence-structure-function interactions, and allowing the generation of new sequences fulfilling multiple goals at the same time (Koh et al., 2025).

Protein design is especially applicable in biological processes in livestock systems with regard to immunity, metabolism, and physiological regulation. Patterns underlying protein stability, interaction potential, and functional diversity can be learned with generative models to help them generate candidate sequences consistent with particular biological objectives. This type of computational exploration allows identifying promising designs more efficiently and eliminates the need to use expensive experimental trial-and-error processes. In the context of smart agriculture, the practices sustain proactive and system-based plans that ensure resiliency and lessen reliance on imported inputs (Stocco et al., 2025).

Designing gene sequences expands these functions to the importance of genetic structures and regulators. Coding sequences are not the sole determinants of gene expression, as regulatory architectures that define the place and time of gene expression are also influential. The generation of these architectures can be modeled with generative AI, where the changes of sequences and their impact on regulatory behavior are learned, and gene constructs with custom expression profiles are designed. This facilitates more specific genetic interventions in animal genomics that are consistent with physiological and environmental conditions, which is critical to sustainable agricultural innovation (Zhou et al., 2026).

They are also used in genome editing to optimize strategies by generating approaches to genome editing that model sequence characteristics related to the efficiency and specificity of editing. Genetic modifications can be more reliable and targeted through the assistance of generative AI by exploring alternative designs in silico. Nevertheless, the shift in the computational design to the biological implementation has to be well-verified because the generated sequences have to work within the functioning of complex living systems. The scope and objective of genetic design also bring out some ethical considerations underpinning the significance of responsible governance in genetic design (Li et al., 2025).

Despite the rapid advancement of generative models for designing protein and gene sequences, a critical bottleneck remains in the form of biological hallucinations. These are generated sequences that appear statistically plausible yet fail to exhibit functional or structural validity in biological systems. Although modern generative architectures can explore vast regions of sequence space and produce novel sequences with desired computational properties, accurately predicting protein folding, stability, and physiological activity in vivo is still very difficult. This reflects the complexity of biological systems, where functional outcomes depend on primary sequence information, higher-order structural dynamics, cellular context, and environmental interactions. Consequently, sequences generated in latent space may not reliably translate into viable biological entities, even when they satisfy model-based optimization criteria. Closing this gap requires tighter integration of generative modeling, structural biology, and experimental validation frameworks, as well as the development of hybrid approaches that incorporate biophysical constraints and functional screening. Therefore, recognizing and mitigating biological hallucinations is essential for advancing generative AI from theoretical sequence design to practical applications in animal genomics and biotechnology (Winnifrith et al., 2024; Zhu et al., 2024b). Generative AI methods for protein, gene, and regulatory sequence design in livestock systems are listed in Table 9.

**Table 9:** Generative AI methods for protein, gene, and regulatory sequence design in livestock systems.

Design target	Biological objective	Generative AI approach	Expected benefits	Key limitations and considerations
Protein sequence design	• Improve protein function and stability • Enhance biological activity relevant to livestock traits	• Learns protein sequence distributions • Generates novel sequences optimized for functional constraints	• Accelerates discovery of functional proteins • Supports in silico exploration of large sequence space	• Functional predictions may not translate in vivo • Requires experimental validation
Immune-related protein engineering	• Enhance immune responsiveness and disease resistance • Modulate host-pathogen interactions	• Generates immune protein variants guided by learned functional patterns • Explores sequence diversity under biological constraints	• Supports the development of disease-resilient livestock • Enables proactive health management	• Immune effects are context-dependent • Risk of unintended immunological consequences
Vaccine antigen design	• Optimize antigenicity and immune recognition • Improve vaccine effectiveness	• Generates candidate antigen sequences with predicted immunogenic properties • Simulates epitope diversity	• Reduces trial-and-error in antigen discovery • Speeds vaccine development pipelines	• Immunogenicity predictions are uncertain • Regulatory approval pathways are complex
Gene sequence design	• Optimize gene expression and functionality • Improve genetic efficiency	• Generates coding sequences optimized for expression constraints • Explores alternative gene architectures	• Enhances precision in genetic engineering • Supports functional genomics studies	• Over-optimization may reduce robustness • Contextual regulatory effects may be overlooked
Regulatory element design	• Control gene expression timing and magnitude • Fine-tune regulatory responses	• Models regulatory grammar • Generates promoters and enhancers with desired activity profiles	• Advances understanding of gene regulation • Enables targeted expression control	• Regulatory activity is tissue- and environment-specific • Validation remains challenging
CRISPR target and guide design	• Improve genome editing efficiency and specificity • Minimize off-target effects	• Generates optimized guide sequences under sequence and structural constraints • Evaluates alternative target configurations	• Increases precision of genome editing • Reduces experimental costs	• Off-target prediction is imperfect • Ethical and regulatory considerations apply
Multi-objective sequence optimization	• Balance multiple functional constraints simultaneously • Avoid trade-offs between traits	• Conditions generation on multiple objectives • Explores Pareto-optimal sequence solutions	• Supports holistic biological design • Aligns with sustainability goals	• Increased model complexity • Interpretation of trade-offs may be difficult
Exploration of hypothetical sequence space	• Investigate unobserved but plausible biological designs • Test biological hypotheses	• Generates novel sequences beyond natural observations • Enables in silico experimentation	• Expands biological knowledge frontier • Supports innovation in livestock genomics	• Hypothetical designs may lack feasibility • Requires conservative application

### 3.4 Enhancing genomic selection and trait prediction

Improving genomic selection and prediction of traits is one of the key uses of generative AI in animal genomics, with a direct impact on breeding choices and the overall performance of the system in the long term. Genomic selection relies upon the ability to infer a precise association between genetic data and phenotypic measurement, which is complicated by the complex structure of the traits under analysis, interaction between the environment and the phenotype, and data constraints. Generative AI builds upon more traditional predictive models because it allows data augmentation, describes latent processes, and incorporates heterogeneous information (Pérez-Enciso et al., 2025; Venegas et al., 2025). Unlike traditional genomic prediction models, which estimate breeding values based on observed data, generative approaches can simulate new genotype-phenotype combinations. This offers expanded possibilities for exploring genetic variation and breeding strategies (Pérez-Enciso et al., 2025).

Synthesis of synthetic phenotypic data, especially of rare or hard-to-measure traits, is one of the most influential applications of generative AI to genomic selection. Generative models can be used to scale up sparse data and make models more robust by learning jointly distributed genomic and phenotypic features. This has the advantage of propelling more credible predictions in the face of uncertainty, which is essential in smart farming systems to allow them to function under a wide range of circumstances (Pérez-Enciso et al., 2025).

Trait prediction can also be improved with generative models, which can learn non-linear and higher-order interactions that underlie complex phenotypes. These models can capture shared biological aspects that affect a variety of traits in the form of latent representations, which can be used to predict many traits and conduct a holistic assessment of breeding goals. These representations are additionally integrated with multi-omics data to establish a connection between genetic variation and downstream molecular processes. Combined, these capabilities make it possible to have more biologically based and flexible genomic selection strategies (Lee et al., 2024). Despite these developments, one must be keen on assessing developments in predictive performance to determine whether they are true biological observations or due to synthetic data or model complexity. The issue of interpretability is also a major challenge; stakeholders need to know how predictions are made to rely on them and be able to put them into practice. These issues need to be addressed to bring about generative AI-enhanced genomic selection to real-world smart agricultural applications (Pérez-Enciso et al., 2025).

Advances in high-throughput phenotyping technologies are enabling the integration of genomic and phenotypic data by generating detailed and continuous representations of animal morphology, physiology, and behavior. Often based on imaging systems, depth sensing, and automated monitoring platforms, these technologies provide rich, multidimensional phenotypic descriptors that extend far beyond traditional trait measurements. Consequently, phenotypic data is becoming more granular, dynamic, and context-dependent, capturing subtle variations that reflect underlying biological processes and environmental influences. This transformation is reshaping the landscape of animal genomics by expanding the range and resolution of observable traits that can be linked to genetic variation (Upadhyay et al., 2024).

In this context, integrating phenomic and genomic data is essential for understanding genotype-phenotype relationships. Traditional genomic analyses have relied on relatively coarse or static phenotypic measurements, which limit the ability to detect complex genetic effects and interactions. In contrast, high-resolution phenotypic data derived from advanced sensing technologies allows for a more precise characterization of traits and their temporal dynamics. Combining these data with genomic information supports more comprehensive analytical frameworks that capture genetic determinants and their expression under varying environmental conditions. This integration is especially important in livestock systems, where phenotypes are affected by genetic, management, and environmental factors (Shakshi et al., 2024; Upadhyay et al., 2024).

Generative AI is a powerful approach for integrating heterogeneous data sources and learning joint representations that capture the relationships among genomic variation, phenotypic expression, and contextual factors. Through latent variable modeling and multimodal learning, generative models synthesize information across data types. This enables the reconstruction and simulation of phenotypic outcomes from genomic inputs and vice versa. This capability allows for more precise and flexible modeling of complex traits, including those that are challenging to measure directly or that change dynamically over time. Furthermore, generative approaches facilitate exploring hypothetical scenarios, allowing researchers to investigate how changes in genomic or environmental conditions could influence phenotypic outcomes (Kuznets-Speck et al., 2025).

Integrating high-throughput phenotyping with genomics strengthens the empirical basis of genomic analyses by providing richer datasets for validation and interpretation. Linking detailed phenotypic descriptors to genomic markers allows for the more precise identification of genetic associations and improves the interpretability of predictive models. The convergence of phenomics and genomics reflects a shift toward more data-intensive, integrative approaches in animal science, where data collection, modeling, and application are increasingly interconnected (Choudhary et al., 2024). Generative AI is a key enabler of this transition, providing tools that can manage and extract insights from complex, multimodal datasets. Additionally, recent studies have shown that high-resolution phenotypic traits obtained from three-dimensional imaging systems can be quantitatively linked to genomic variation via genome-wide association frameworks. This illustrates the feasibility of integrating computer vision–based phenotyping with genetic analysis. These approaches exemplify how complex morphological features, captured via depth-sensing technologies, can serve as informative phenotypic inputs for genomic modeling. This reinforces the value of integrating multimodal data to advance genotype-phenotype inference (Ruchay et al., 2022).

From a smart agriculture perspective, integrating genomic and high-throughput phenotypic data supports developing more responsive and adaptive livestock management systems. These systems capture real-time phenotypic variation and link it to genetic potential, informing dynamic decision-making processes that optimize productivity, health, and welfare. Generative models' ability to incorporate and synthesize such data enhances their role in predictive and prescriptive analytics, providing a more comprehensive understanding of animal systems. Thus, this multimodal integration represents a critical step toward realizing the full potential of generative AI in animal genomics and its application in intelligent, sustainable agricultural systems (Santhosh, 2024).

Despite growing interest in generative AI approaches for genomic selection and complex trait prediction, traditional methods, particularly linear mixed models, such as genomic best linear unbiased prediction (GBLUP), remain highly optimized and validated across livestock species. These models have strong theoretical foundations, are computationally efficient, and have been extensively validated in breeding programs. This makes them robust benchmarks for evaluating newer approaches (Bermann, 2022). In contrast, generative models are in the early stages of application in this field, and their advantages over established methods have not been consistently demonstrated under practical conditions. While generative frameworks have the potential to capture complex, nonlinear relationships and integrate heterogeneous data sources, their performance relative to well-established statistical approaches is context-dependent and requires further systematic evaluation. Therefore, the current evidence indicates that generative AI should be considered a complement to, rather than a replacement for, traditional genomic prediction models, especially in operational breeding settings where reliability and interpretability are essential (Džermeikaitė et al., 2025). Contributions of generative AI to genomic selection and complex trait prediction are shown in Table 10.

**Table 10:** Contributions of generative AI to genomic selection and complex trait prediction.

Application aspect	Genomic selection or trait context	Generative AI contribution	Advantages for prediction accuracy	Limitations and cautionary notes
Synthetic phenotype generation	• Rare, low-heritability, or difficult-to-measure traits • Sparse phenotypic records	• Generates plausible synthetic phenotypes conditioned on genomic patterns • Expands effective training data	• Improves learning for underrepresented traits • Reduces sensitivity to missing data	• Synthetic traits may not capture full biological variability • Requires careful validation
Augmented training for genomic prediction models	• Breeding value estimation • Polygenic trait prediction	• Supplies additional realistic genotype-phenotype pairs • Regularizes model training	• Enhances robustness and generalization • Reduces overfitting in small datasets	• Performance depends on the quality of the generated data • Bias amplification is possible
Modeling non-linear genetic effects	• Epistasis and complex gene interactions • Non-additive trait architectures	• Learns joint distributions capturing non-linear dependencies • Simulates interaction effects	• Improves prediction of complex traits • Moves beyond additive assumptions	• Interpretability of learned interactions is limited • Validation is challenging
Prediction under data imbalance	• Unequal representation of breeds, lines, or environments • Skewed training datasets	• Generates balanced synthetic samples across groups • Smooths uneven data distributions	• Reduces population-specific bias • Improves the fairness of predictions	• May obscure true biological differences • Requires population-aware constraints
Multi-omics-enhanced trait prediction	• Traits influenced by regulatory and molecular layers • Complex biological pathways	• Integrates genomic data with simulated or inferred multi-omics signals • Learns cross-layer dependencies	• Enhances biological realism • Improves prediction of context-dependent traits	• Multi-omics data scarcity • Increased model complexity
Uncertainty-aware prediction	• Traits with variable expression across environments • Management-sensitive outcomes	• Produces probabilistic predictions rather than point estimates • Quantifies uncertainty	• Supports risk-aware selection decisions • Improves decision confidence	• Uncertainty estimates may be misinterpreted • Calibration is required
Cross-population prediction transfer	• Prediction in new breeds or environments • Limited local training data	• Leverages learned distributions to adapt predictions across populations • Supports transfer learning	• Extends applicability of genomic selection models • Supports inclusive breeding programs	• Transferability depends on genetic similarity • Risk of reduced accuracy
Scenario-based selection simulation	• Evaluation of alternative breeding strategies • Long-term genetic outcomes	• Simulates trait trajectories under hypothetical selection scenarios • Explores future outcomes	• Enables strategic planning • Supports sustainability-oriented selection	• Simulations may oversimplify biological and environmental dynamics

### 3.5 Understanding gene regulation and epigenomics

The field of gene regulation and epigenomics is essential in bridging genomic variation to phenotypic expression, and it is naturally an area where generative AI can be applied. The regulatory mechanisms exist on multiple layers of molecules and are highly context-dependent, which makes it problematic to apply standard methods of analysis. Generative models provide a versatile structure for learning regulatory grammar and simulating the interaction of sequence and epigenetic features to regulate the expression of genes (DaSilva et al., 2025). Generative models have a clear advantage in that they enable the simulation of regulatory landscapes under hypothetical conditions, which cannot be directly inferred through predictive modeling alone.

By simulating chromatin accessibility and epigenomic landscapes, generative AI can be used to explain regulatory states that can control biological functionality. Such models can learn the variations in the regulatory patterns among different contexts and synthesize plausible regulatory patterns under varying conditions. Such modeling in smart agriculture assists in predicting phenotypic responses to changes in the environment and management (Mirkute, 2025).

Direct prediction of gene expression using sequence is also made easy through generative methods, which model distributions of potential expression outcomes as opposed to single-point estimates. This risk-conscious view of the world is one that is consistent with biological reality. Relevance is further improved by context-specific modeling, which models tissue or condition-dependent regulatory behavior (Ramakrishnan et al., 2025).

The problems are not yet overcome in the availability of data, biological validation, and integration of mechanistic knowledge. Livestock species have regulatory annotations that are not complete enough to raise more doubts about model outputs. These shortcomings can be overcome by proper curation of data and integration of biological priori. However, generative AI offers a strong blueprint for propelling regulatory genomics in intelligent agriculture (Gusev et al., 2025). Generative modeling of gene regulation and epigenomic landscapes is presented in Table 11.

**Table 11:** Generative modeling of gene regulation and epigenomic landscapes.

Regulatory or epigenomic focus	Biological scope	Generative modeling role	Insights and applications enabled	Limitations and key considerations
Chromatin accessibility modeling	• Open and closed chromatin states • Regulatory potential of genomic regions	• Learns distributions of accessibility patterns • Generates plausible chromatin state profiles	• Improves understanding of regulatory potential • Supports prediction of condition-dependent gene activity	• Accessibility is cell- and context-specific • Limited availability of high-resolution data
DNA methylation pattern generation	• Epigenetic modification affecting gene regulation • Stable and dynamic methylation states	• Models methylation landscapes across genomic regions • Simulates context-dependent methylation changes	• Enables inference of regulatory repression or activation • Supports epigenome-aware trait prediction	• Methylation effects are highly tissue-dependent • Temporal dynamics are difficult to capture
Histone modification modeling	• Post-translational histone marks • Chromatin state regulation	• Generates combinatorial histone mark patterns • Captures co-occurrence structures	• Advances the interpretation of regulatory states • Improves functional annotation of non-coding regions	• Sparse and heterogeneous datasets • Complex mark interactions challenge validation
Enhancer and promoter design	• Regulatory elements controlling transcription • Sequence-level regulatory grammar	• Learns sequence-regulation relationships • Generates candidate regulatory elements with desired activity	• Supports targeted modulation of gene expression • Enhances regulatory hypothesis testing	• Activity depends on cellular context • Functional validation remains essential
Gene expression prediction from sequence	• Transcriptional output driven by regulatory architecture • Sequence-dependent expression	• Models mapping from DNA sequence to expression distributions • Simulates expression variability	• Improves prediction of functional impact of variants • Supports integration of genomics and transcriptomics	• Expression influenced by many non-sequence factors • Risk of oversimplification
Cell-type or tissue-specific regulation	• Context-dependent regulatory programs • Differential gene activity	• Generates regulatory profiles conditioned on cellular context • Learns tissue-specific patterns	• Enables context-aware genomic interpretation • Improves precision of trait modeling	• Requires well-annotated context labels • Limited generalizability across tissues
Integration of regulatory layers	• Interaction among chromatin, methylation, and transcription • Multi-layer regulation	• Jointly models multiple epigenomic modalities • Captures cross-layer dependencies	• Provides a holistic view of gene regulation • Enhances biological realism	• High model complexity • Data alignment and normalization challenges
Simulation of regulatory perturbations	• Effects of genetic or epigenetic changes • Regulatory system response	• Simulates regulatory consequences of hypothetical perturbations • Explores system dynamics	• Supports in silico experimentation • Aids interpretation of variant effects	• Perturbation effects may be inaccurately estimated • Requires conservative interpretation

### 3.6 Applications in precision breeding and reproductive technologies

Generative AI has become a more significant part of precision breeding and reproductive technologies by increasing the value of translating genomic knowledge into reproductive decisions. Reproductive processes are regulated by complex genetic and developmental factors, and information in this sphere is usually scarce and skewed. Generative models can be used to fill in thin datasets, learn latent predictors of reproductive success, and enable predictive and simulation-based methods (Haider et al., 2025). This reflects a shift from prediction to design. In this new approach, generative frameworks support the conceptualization of optimized genetic configurations, rather than merely forecasting the outcomes of existing ones.

By simulating the relationship between genetic factors and developmental states to predict reproductive outcomes, generative AI can improve the quality of evaluation of embryos and gametes. Additionally, synthetic data generation helps in training and validating predictive models, as it enables making sound and objective decisions about reproduction. The capabilities will lead to a more effective utilization of reproductive resources and a better correlation between the goal of selection and reproductive implementation (Selntigia et al., 2025). The design and optimization of reproductive strategies are also supported by generative AI, as it allows simulating alternative scenarios before implementation. This predictive ability is in line with the proactive approach to management and long-term planning of smart agriculture. The questions of ethics associated with welfare, diversity, and governance are kept in focus, which explains the necessity of transparency and sound use (Shao et al., 2025). Applications of generative AI in precision breeding and reproductive technologies are outlined in Table 12.

**Table 12:** Applications of generative AI in precision breeding and reproductive technologies.

Application domain	Biological or reproductive focus	Generative AI function	Benefits of precision breeding	Limitations and considerations
Embryo viability assessment	• Early developmental potential • Genetic and epigenetic quality	• Generates synthetic representations of embryo states • Learns patterns associated with viability	• Improves selection accuracy • Reduces reliance on invasive or subjective assessments	• Viability signals are multifactorial • Risk of overconfidence without validation
Sperm quality modeling	• Motility, morphology, and functional competence • Genetic integrity	• Learns distributions of sperm characteristics • Generates representative quality profiles	• Enhances screening efficiency • Supports objective and scalable assessment	• Quality traits vary with environment and handling • Data heterogeneity challenges
Oocyte quality evaluation	• Developmental competence and maturation state • Epigenetic readiness	• Models latent features associated with oocyte quality • Simulates quality variation	• Improves reproductive efficiency • Supports informed mating decisions	• Limited high-quality training data • Context-specific biological effects
Assisted reproductive technology optimization	• In vitro fertilization and embryo transfer outcomes • Protocol sensitivity	• Simulates outcomes under alternative protocols • Explores parameter combinations	• Enables protocol refinement • Reduces trial-and-error experimentation	• Simulated outcomes may oversimplify biology • Requires careful calibration
Mating and crossing strategy design	• Genetic complementarity and diversity • Long-term breeding goals	• Generates hypothetical mating scenarios • Evaluates projected genetic outcomes	• Supports strategic and sustainable breeding plans • Balances gain and diversity	• Long-term predictions carry uncertainty • Ethical and management constraints apply
Selection of donors and recipients	• Compatibility between genetic and physiological profiles • Reproductive success likelihood	• Models joint distributions of donor-recipient traits • Simulates pairing outcomes	• Improves success rates of reproductive interventions • Supports individualized decisions	• Requires comprehensive phenotypic data • Risk of hidden confounding factors
Reproductive trait prediction	• Fertility, gestation success, and reproductive longevity • Complex trait architecture	• Generates probabilistic predictions of reproductive performance • Captures non-linear effects	• Enhances early selection decisions • Supports risk-aware breeding	• Prediction uncertainty may be underestimated • Interpretability remains limited
Integration with precision livestock systems	• Real-time reproductive monitoring • Management-driven interventions	• Synthesizes genomic and sensor-derived reproductive data • Supports adaptive decision-making	• Enables responsive and individualized reproductive management • Improves overall system efficiency	• Data integration complexity • Dependence on infrastructure quality

### 3.7 Cross-species genomic modeling

Cross-species genomic modeling is an important strategic use of generative AI that can solve data imbalance and contribute to agricultural innovation inclusively. Generative models have the potential to apply information in resource-rich species to other species with limited resources by learning transferable representations of genomic structure and function. This transfer learning feature provides comparative genomics and improves the scope of genomics-impaired agriculture (Tapp et al., 2025). In this context, generative models facilitate the transfer and synthesis of genomic patterns across species. This extends beyond predictive transfer because it enables the creation of new representations adapted to populations with limited data.

Generative AI allows studying both conserved and divergent genomic properties of species, in terms of hypothesis generation and inference of functions. By combining multi-omics data with modeling, cross-species modeling can be further enhanced or enriched to reflect the similarities in regulatory or expression characteristics. Such abilities are especially useful in intelligent farming systems that include a variety of species and production environments (Huang et al., 2025).

The most important issue is to balance between generalization and sensitivity to species-specific characteristics. The danger of overgeneralization is the blurring of essential biological differences, whereas over-specialization restricts transferability. To cope with these challenges, sharply prescribed models should be designed, and evolutionary principles should be taken into consideration. Tactfully used, cross-species generative modeling will help create resilient, adaptable, and diverse smart agriculture (Tapp et al., 2025).

One particularly promising approach to cross-species genomic modeling is applying transfer learning to address disparities in data availability across livestock populations. In this context, models trained on data-rich species or highly characterized commercial breeds can be adapted to data-limited or indigenous populations through fine-tuning or domain adaptation strategies. These approaches allow for the transfer of learned genomic representations, such as sequence patterns, regulatory features, and genotype-phenotype relationships. This reduces dependence on large-scale labeled datasets in underrepresented populations. Although biological differences between breeds and species present challenges related to domain shift and model generalization, transfer learning frameworks provide a practical way to extend the advantages of advanced generative models to various agricultural systems. This paradigm is particularly relevant for smart agriculture, where improving genomic prediction and breeding strategies in locally adapted or resource-constrained environments is essential for sustainability and resilience (Latyshev et al., 2023; Pillai et al., 2025). Cross-species and transfer learning strategies enabled by generative AI are shown in Table 13.

**Table 13:** Cross-species and transfer learning strategies enabled by generative AI.

Strategy type	Biological scope	Generative AI mechanism	Benefits of genomic analysis	Limitations and risks
Transfer learning from data-rich species	• Well-characterized livestock species • Extensive genomic resources	• Pretrains generative models on large datasets • Fine-tunes on target species data	• Reduces data requirements for understudied species • Accelerates model convergence	• Risk of bias toward source species • Requires careful adaptation
Cross-species sequence representation learning	• Conserved genomic and regulatory elements • Evolutionary constraints	• Learns shared latent representations across species • Captures conserved sequence patterns	• Improves functional annotation • Enables comparative genomics	• Species-specific features may be underrepresented • Interpretability challenges
Synthetic data generation for understudied species	• Rare, indigenous, or minor livestock species • Sparse genomic datasets	• Generates plausible synthetic genomes guided by related species • Expands effective training data	• Supports genomic research in data-poor contexts • Aids conservation and inclusion	• Synthetic data may not fully reflect unique adaptations • Validation difficulty
Domain adaptation across production systems	• Same species across different environments or management systems • Environmental heterogeneity	• Adjusts learned distributions to new domains • Models context-dependent genomic effects	• Enhances robustness of predictions • Improves applicability across systems	• Environmental confounding may persist • Requires domain-aware constraints
Comparative regulatory modeling	• Regulatory architectures across species • Non-coding genome	• Learns shared and divergent regulatory patterns • Generates cross-species regulatory hypotheses	• Advances understanding of regulatory evolution • Improves regulatory variant prediction	• Regulatory logic is highly context-specific • Sparse comparative epigenomic data
Multi-species foundation models	• Broad taxonomic coverage • Diverse genomic sequences	• Trains large generative models on pooled multi-species data • Enables zero- or few-shot learning	• Facilitates rapid deployment across species • Supports scalable smart agriculture solutions	• High computational cost • Risk of overgeneralization
Cross-breed knowledge transfer	• Breeds within a species with uneven data availability • Genetic diversity	• Transfers learned patterns across breeds • Generates balanced representations	• Improves prediction in minority breeds • Reduces breed-specific bias	• May smooth over meaningful breed differences • Requires population-aware evaluation
Evolution-informed generative modeling	• Phylogenetic relationships • Adaptive divergence	• Constrains generation using evolutionary priors • Models divergence and conservation jointly	• Enhances biological plausibility • Supports long-term sustainability analysis	• Evolutionary assumptions may be oversimplified • Increased modeling complexity

## 4 Integration of generative AI into smart agriculture systems

The introduction of generative AI into intelligent systems of agriculture is a key move towards handling the increasingly complex nature of livestock production in a data-intensive and biologically informed way. The idea of smart agriculture is aimed at integrating digital technologies, the understanding of biology, and dynamic decision-making to enhance productivity, fortitude, and sustainability in uncertain conditions. In this paradigm, generative AI is a source of modeling frameworks with learning latent structures, uncertainty, and information synthesis by heterogeneous sources of data. When used in animal genomics, these features permit the translation of molecular data to system-level data useful in intelligent livestock management. The potential of generative AI integration is thus not restricted to the analysis improvement but essentially transforms the process of biological potential, environmental conditions, and management behavior connectivity in the smart agricultural systems (Min et al., 2025; Robben, 2025). Integration of generative AI with smart agriculture systems and precision livestock farming is presented in Table 14.

**Table 14:** Integration of generative AI with smart agriculture systems and precision livestock farming.

Integration layer	System components involved	Role of generative AI	Operational benefits	Challenges and constraints
Genomics-phenotype integration	• Genomic profiles • Phenotypic and performance records	• Learns joint distributions linking genomes to phenotypic outcomes • Generates predictive phenotypic scenarios	• Improves individual-level prediction accuracy • Supports personalized livestock management	• Phenotypes are environment-sensitive • Data incompleteness affects reliability
Precision livestock farming platforms	• Sensor networks • Automated monitoring systems	• Synthesizes genomic data with real-time sensor inputs • Generates context-aware management recommendations	• Enables adaptive and timely interventions • Enhances animal health and welfare monitoring	• Data heterogeneity and noise • Dependence on infrastructure quality
Decision support systems	• Farm management software • Advisory tools	• Simulates alternative decision pathways • Generates outcome-aware recommendations	• Supports proactive and evidence-based management • Reduces uncertainty in complex decisions	• Model outputs may be difficult to interpret • Risk of over-reliance on AI suggestions
Digital twins frameworks	• Virtual animal representations • Longitudinal data streams	• Generates dynamic simulations of biological states • Updates predictions with incoming data	• Enables predictive and scenario-based management • Supports long-term planning	• High computational demands • Validation of simulations is challenging
Breeding and selection pipelines	• Genomic selection systems • Breeding program databases	• Generates synthetic data to augment selection models • Simulates genetic trajectories	• Improves robustness of selection decisions • Balances genetic gain and diversity	• Synthetic data may introduce bias • Long-term effects are uncertain
Environmental and management context modeling	• Climate data • Feeding and housing conditions	• Generates environment-conditioned biological responses • Models genotype-environment interactions	• Enhances resilience to environmental variability • Supports sustainable management strategies	• Environmental data integration is complex • Contextual generalization is limited
Sustainability and resource optimization	• Resource use metrics • Environmental impact indicators	• Simulates long-term system outcomes under alternative strategies • Explores trade-offs	• Supports sustainability-oriented decision-making • Aligns productivity with environmental goals	• Outcomes depend on model assumptions • Requires cautious interpretation
Farm-to-system-level coordination	• Individual farms • Regional or national agricultural systems	• Aggregates and generates system-level insights from distributed data • Supports coordinated planning	• Improves scalability of smart agriculture • Enables policy-relevant analysis	• Data governance and interoperability challenges • Privacy and ownership concerns

### 4.1 Associating genomics with precision livestock farming (PLF)

The connection between genomics and precision livestock farming is an essential merger of both long-term information on biology and real-time data on management. Precision livestock farming is founded on a sustained observation of animals by using digital devices that intercept physiological, behavioral, and environmental cues to provide responsive management to enhance efficiency, well-being, and productivity. To the extent, however, that the majority of precision livestock farming systems are run at the phenotypic level, and can react to observational conditions but not to predicted biological possibilities. The addition of genomic information adds a fixed and inheritable level of knowledge that represents underlying predispositions, which determine the responses of animals to the management and environmental conditions. This integration makes smart agricultural systems shift to proactive and predictive adjustment as opposed to reactive adjustment (Nsabiyeze et al., 2025; Zhu et al., 2025).

Genomic information gives an understanding of inherent ability and limitations that an animal needs to maintain over its lifetime, whereas phenotypic information that is captured by precision livestock farming technologies represents dynamic and often temporary conditions. By combining these layers, it is possible to make observations in real-time more nuanced, as they will be placed into the genetic background of an animal. This is achievable with the help of generative AI that learns joint representations between some static genomic features and time-varying phenotypic cues. With these models, variation that is observed can be interpreted as the outcome of interactions between genetic predisposition and conditions of the present, rather than as isolated outcomes. This method facilitates a better distinction of the short-term environmental impact and the genetic responses, in addition to increasing the biological significance of management choices (Eckhardt et al., 2025; Zhu et al., 2025). Recent advances in smart dairy farming demonstrate the integration of AI with real-time livestock monitoring systems. Vision-based platforms enable the continuous assessment of animal health, behavior, and productivity through non-invasive sensing and edge-based data processing (Moe et al., 2025).

The importance of the role of generative AI is especially significant since the genomic and precision livestock farming data vary significantly in terms of their structure, scale, and time span. Genomic data are high-dimensional but fairly static, while precision livestock farming data are continuous, multi-modal, and time-rich. Generative models can specifically learn across these disparities by common latent structures, which are used to coordinate heterogeneous sources of data. They do this so that they can allow the formation of integrated representations that bridge the gap between genotype and the reality of performance patterns. These representations are critical to the achievement of the full potential of smart agriculture, since they aid in personalized modeling and adaptive decision-making based on biological knowledge (Ajith, 2024; Gogineni & Mupparaju, 2025).

Using genomics with precision livestock farming based on generative AI, it is possible to manage animals in a more individualized way than simply using averages of the population. Genomic information offers an additional level of individual specificity to the field of precision livestock farming, which already allows individual monitoring, but represents a more profound level of specificity in metabolism, stress response, susceptibility to diseases, and developmental potential. The interaction between these genetic factors and the environmental and management factors, which result in the production of individual phenotypic outcomes, can be learned by generative models. This kind of personalization helps in targeted interventions, minimizes unnecessary inputs, and leads to more efficient and welfare-conscious livestock systems (Eckhardt et al., 2025; Ghavi Hossein-Zadeh, 2025).

On a larger system scale, the connection of genomics and precision livestock farming can lead to the improvement of the strategic capacity of smart agriculture. Instead of maximizing the problems of isolated operational decisions, integrated models may help analyze the management decisions with respect to long-term goals. The generative AI has been used to analyze scenarios to show scenarios of trade-offs between productivity, welfare, and resource use, and to aid informed decisions at both operational and strategic levels. Although data integration, interpretability, and governance still have issues, the integration of genomics and precision livestock farming enabled by generative AI is a first step towards biologically aware intelligent agriculture (Vlaicu et al., 2024).

The integration of generative AI into animal genomics must be understood in the context of the broader transformation of agricultural systems, which is being driven by digital technologies and intelligent infrastructure. Contemporary livestock production is increasingly shaped by the convergence of sensing technologies, automated management systems, and data-driven decision frameworks. These elements collectively form the foundation of precision livestock farming. In this evolving landscape, genomic information is just one part of a highly interconnected system where biological, environmental, and operational data are constantly generated and integrated. Therefore, generative AI–enabled genomic insights achieve their full impact not in isolation, but through their incorporation into this wider technological ecosystem (Silvestri et al., 2026).

Recent developments in digital livestock systems demonstrate the growing complexity of animal housing environments, monitoring platforms, and control mechanisms that enable real-time evaluation of animal health, welfare, and productivity. These systems rely on continuous data streams derived from sensors, imaging technologies, and automated recording devices. Collectively, these data streams provide a dynamic representation of the production environment. Integrating this infrastructure with genomic and multi-omics data creates new opportunities for comprehensive system modeling, enabling the evaluation of genetic potential in conjunction with environmental conditions and management practices. Generative AI plays a critical role in this context by enabling the synthesis and interpretation of heterogeneous data streams, thereby supporting more adaptive and context-aware decision-making processes (Lima & Alves, 2025; Sharma et al., 2025).

Furthermore, the integration of generative AI with digital agricultural infrastructure enables the development of closed-loop systems where data acquisition, model inference, and management actions are seamlessly connected. In these systems, generative models simulate alternative scenarios, anticipate system responses, and inform interventions in near real time. This contributes to more efficient and resilient livestock production. This capability is particularly relevant for addressing complex challenges such as environmental variability, resource optimization, and animal welfare, for which static or isolated analyses are insufficient. By embedding genomic insights within these dynamic systems, generative AI enables the transition from reactive to proactive and predictive management strategies (Santhosh, 2024; Xu et al., 2026).

Integrating genomic intelligence with digital infrastructure raises important considerations regarding system interoperability, data governance, and scalability. Effective implementation requires harmonization of diverse data sources and platforms, as well as robust frameworks for data sharing and model deployment. The increasing automation and complexity of these systems also necessitate careful consideration of ethical, regulatory, and socio-technical factors, especially since decision-making is becoming more distributed among human and machine agents. These challenges highlight the need to view generative AI as more than just a computational tool; it is also a component of a broader socio-technical system that will shape the future of animal agriculture (Hostens et al., 2025; Hatakeyama, 2026).

Contextualizing generative AI within digitally transformed livestock systems offers a clearer picture of its role in advancing smart agriculture. This perspective highlights the interdependence between computational innovation and physical infrastructure and emphasizes that genomic modeling's value is realized through its integration into operational environments. This systems-level perspective underscores the necessity of interdisciplinary approaches connecting genomics, artificial intelligence, engineering, and agricultural sciences. These approaches enable the development of intelligent, sustainable, and responsive livestock production systems (Santhosh, 2024).

### 4.2 Digital twins for livestock

Livestock digital twins represent a new paradigm that expands the application of generative AI and genomics to the dynamic and personalized system representation. An example of a livestock digital twin can be thought of as the virtual shadow of a real animal that would develop under the action of streams of data and computational models. Digital twins, unlike static simulations, are aimed at replicating the current biological processes and interactions between the manager and the animal, as well as providing continuous assessment and prediction of the animal's state. The core concept of this idea is making use of generative AI, which enables the digital twins to embody uncertainty, variability, and non-linear dependencies of the living system (Ghavi Hossein-Zadeh, 2025; Nezzi et al., 2025).

Genomic data serves as a reference point for livestock digital twins, with inherent potential and biological limitations that drive lifelong growth. Phenotypic data vary over time; however, genomic data do not, and thereby allow modeling in an individual way at the beginning. Genetic variation can be combined with observable phenotypes and environmental factors through the use of genomic information combined with phenotypic and environmental data, with the help of generative AI, which learns latent representations between them. It is possible with the help of such integration that digital twins can model the genetic potential development in different conditions, providing personalized and biologically based system representations (Sun et al., 2025).

One major characteristic of digital twins based on generative AI is that they are able to simulate uncertainties and variability instead of making single deterministic predictions. Livestock systems are stochastic in nature, with individuals having varying responses to the same condition. Generative models represent the outcome distributions, allowing digital twins to produce the space of plausible future states. The risk-averse decision-making and probabilistic view is consistent with the biological reality and helps manage smart agriculture, where the risk should be included in management decisions instead of using overconfident prognoses (Mannone et al., 2025).

Digital twins are also integrative platforms that integrate heterogeneous data sources at biological scales. The data of genomics, physiology, behavior, and the environment vary significantly in terms of their resolution and structure, but generative AI can resolve the discrepancies using common latent spaces. This integration allows the representation of animals with digital twins in a holistic fashion as opposed to a collection of measurements that are not connected. These holistic representations have especially been useful in elucidating complex traits and health conditions that arise out of a combination of several biological layers (Métayer et al., 2025).

Digital twins are also adaptive in nature, which increases their suitability in smart agriculture. Generative models can also update the digital twin based on new information: the collected data can be used to change the model to indicate changes in both the state of the animals and the environmental conditions. This ability to have continuous learning will ensure that digital twins are updated and responsive as opposed to being stagnant and outdated models. This adaptability is crucial to decision support in livestock systems, which are dynamic in nature and whose objectives undergo change (Kallenberg et al., 2025).

**Fig. 3:**
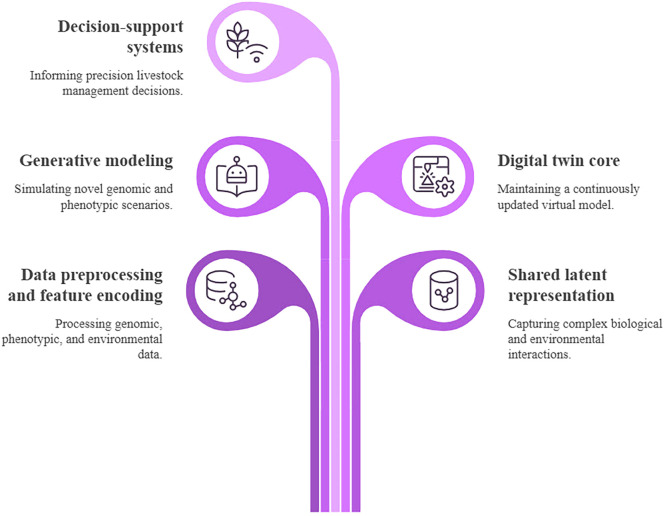
Unveiling the generative AI-driven digital twin framework.

Even with this potential, livestock digital twins face issues in the areas of biological realism, interpretability, and governance. Simulation models can learn correlations not based on causality, and the validity of the simulated results is questionable. To make digital twins biologically meaningful, it is necessary to design models carefully, incorporate domain knowledge, and continuously validate them. Also, generative digital twins may be complex, which may impair interpretability, and it is therefore necessary to create transparent interfaces and explanation aids to foster trust and adoption. The issues in data utilization and decision authority also involve an ethical aspect, which only contributes to the necessity of responsible governance when digital twins become more prominent in smart agriculture systems (Arulmozhi et al., 2024; Subeesh & Chauhan, 2026). Fig. 3 shows the computational architecture of a generative AI-driven digital twin framework for livestock systems. It illustrates how genomic, phenotypic, and environmental data are integrated into a unified modeling pipeline. First, multimodal data streams are processed through preprocessing and feature encoding stages. Then, they are integrated into a shared latent representation that captures complex biological and environmental interactions. Generative modeling components, including transformer-, variational-, and diffusion-based architectures, enable the simulation of novel genomic and phenotypic scenarios, supporting dynamic system representation. The digital twin core continuously updates the virtual model of the animal or production system by incorporating observed and generated data to estimate system states and predict future outcomes. This framework's outputs inform decision-support systems for precision livestock management, and feedback loops enable iterative model refinement based on real-time data streams.

### 4.3 From genomic information to management decisions: integrated predictive pipelines

**Fig. 4:**
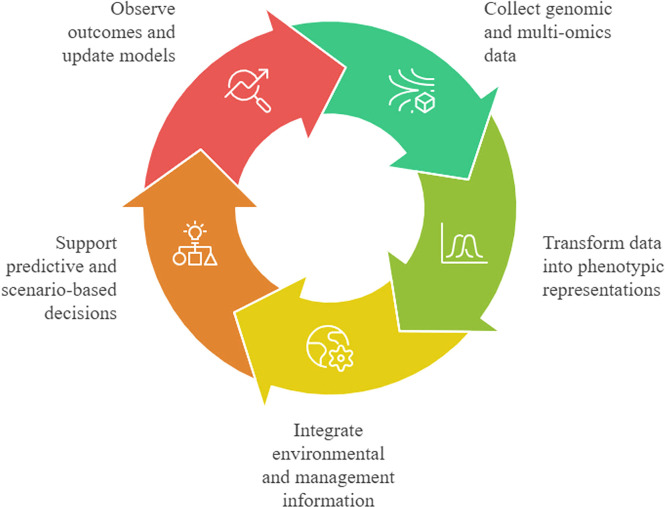
Generative AI-enabled genotype-phenotype-management pipeline.

The working core of smart agriculture is made up of integrated pipelines that bridge the gap between genomic knowledge and phenotypic awareness and management choices. These pipelines describe the relationship between the information on a molecular level and observed features, and finally, to actionable interventions. Historically, genomics, phenotypic surveillance, and managerial decision-making have been regarded as separately related processes. Generative AI allows its combination into end-to-end pipelines that are coherent and capture the complexity and uncertainty of biological systems (Shaikh et al., 2025). Fig. 4 shows the detailed steps of a generative AI-powered pipeline that combines genomic data with phenotypic and environmental information to improve livestock management. The pipeline starts with acquiring multimodal data, such as genomic sequences, sensor-derived phenotypes, and environmental measurements. Next are the preprocessing and feature encoding stages. The inputs are then processed through generative modeling components that learn joint representations and enable the simulation of novel genotype-phenotype relationships or the generation of synthetic data. The outputs are integrated into predictive and decision support systems for applications such as genomic selection, health monitoring, and precision breeding strategies. Feedback loops between real-time farm data and model outputs enable continuous system updating and refinement, supporting adaptive, data-driven livestock management.

In the genomic stage, the information captured passes potential but never directly establishes the results. Phenotypes are the result of interactions between genetic predisposition, dynamic biological, and environmental processes. Generative AI allows us to model this transition by learning probabilistic relationships between genomic variation and phenotypic distributions rather than making deterministic predictions. This method recognizes biological variability and is more realistic in its modeling of the expression of genetic potential with time (Tsuru & Furusawa, 2025).

Precision livestock farming technologies propose phenotypic data that serves as an ongoing feedback source that improves genomic-to-phenotype mappings. Generative models combine these data and genomic priors so that the model can be updated adaptively as new observations are available. This active system sustains the pipeline that is dynamically changing with time and keeps its relevance due to the evolution of animals and changes in the conditions. The outcome is a system that has the capability of continuously learning, rather than using fixed models (Zhu et al., 2025).

The last phase of the pipeline transforms phenotypic knowledge into management decisions. The translation is assisted by generative AI, which approximates how other management strategies will affect future outcomes based on genomic and phenotypic context. This is the ability to make proactive and personalized decisions about the objectives of smart agriculture. The use of pipelines, where results are distributed, can also facilitate the analysis of trade-offs and uncertainty, which enables stakeholders to determine robust strategies in varying circumstances (Arya & Lin, 2025).

Difficulties still exist in providing transparency, data alignment, and ethical governance in integrated pipelines. Generative models are complicated and may cause confusion in causality, which is a major requirement of successful implementation. Strong data infrastructure and governance structures are also needed to ensure that it is deployed responsibly and effectively. However, an illustrative example of this change towards systems-level thinking made possible by generative AI in smart agriculture is integrated predictive pipelines (Chen et al., 2025).

### 4.4 Implications for sustainable agriculture

The possibility of generative AI applications to animal genomics has far-reaching consequences for sustainable agriculture by transforming the control of biological complexity in the long run. Sustainability involves environmental stewardship, financial sustainability, animal welfare, and system resilience, and all these requirements are based on informed and adaptive decision-making. Because genomic innovation can be aligned with long-term system goals rather than short-term optimization through probabilistic modeling, scenario exploration, and integration across biological and operational layers, generative AI can improve sustainability (Pasupuleti, 2025; Robben, 2025).

Generative AI also helps to utilize resources more efficiently and harm the environment less by enhancing the consistency between management procedures and genetic potential. It also contributes to resilience by allowing the exploration of a wide range of genetic and phenotypic conditions, promoting strategies that focus on resilience in the face of uncertainty. The introduction of genomics to system-level modeling also enables an analysis of both environmental and welfare performance and productivity to enable a balanced decision (Berthelot et al., 2025).

Meanwhile, sustainable development is based on responsible management, openness, and participation of stakeholders. Generative AI enhances the strength of genomic decision-making, and it is more important that ethical management is realized and consistent with social values. Building capacity and interpretability is necessary to ensure that advanced modeling tools are deployed effectively and responsibly. Finally, generative AI can be used to reshape animal genomics to enable a shift towards productive, resilient, and sustainable livestock systems (Macha et al., 2024).

## 5 Challenges and limitations

The use of generative AI in animal genomics in a smart agriculture system has considerable opportunities but faces considerable challenges and restrictions, which influence its credibility, equity, and sustainability. These problems are caused by the inherent complexity of biological systems, agricultural data organization and regulation, and the generative model's computational and social environments of their creation and application. In contrast to the domains in which the amount and standardization of data are high, livestock genomics is provided in non-homogeneous, deteriorating, and cost-limited conditions. With the growing use of generative AI in the process of biological interpretation and management decision-making, it is critical to tackle these issues to help technological innovation facilitate sustainable, responsible, and equitable agricultural transformation instead of exacerbating current restraints (Ampatzidis et al., 2025; Pasupuleti, 2025). Major challenges and limitations of generative AI in animal genomics are shown in Table 15.

**Table 15:** Major challenges and limitations of generative AI in animal genomics.

Challenge category	Underlying causes	Impact on genomic modeling	Implications for smart agriculture	Potential mitigation directions
Data scarcity and imbalance	• Limited sample sizes in many species and breeds • Uneven representation across populations	• Reduced model generalization • Overfitting to dominant datasets	• Unequal performance across production systems • Risk of excluding minor or local breeds	• Data augmentation and transfer learning • Coordinated data-sharing initiatives
Data quality and heterogeneity	• Inconsistent data collection protocols • Noise and missing values in genomic and phenotypic records	• Degraded training stability • Unreliable generative outputs	• Reduced trust in AI-driven recommendations • Increased operational uncertainty	• Standardization of data pipelines • Robust preprocessing and quality control
Model bias and fairness	• Historical selection bias • Overrepresentation of high-performing breeds	• Skewed generative outputs • Biased predictions and simulations	• Reinforcement of inequitable breeding practices • Loss of genetic diversity	• Bias-aware training strategies • Population-stratified evaluation
Generalization across contexts	• Strong genotype-environment interactions • Context-specific biological effects	• Poor transferability to new environments or systems • Reduced robustness	• Limited scalability of smart agriculture solutions • Increased deployment risk	• Domain adaptation methods • Environment-conditioned modeling
Computational and resource constraints	• High model complexity • Large training data and energy requirements	• Restricted accessibility to advanced models • Slower iteration cycles	• Barriers for adoption in resource-limited settings • Sustainability concerns	• Model compression and efficiency optimization • Shared computing infrastructure
Validation and biological plausibility	• Difficulty validating synthetic outputs experimentally • Lack of ground truth for generated data	• Uncertainty about biological realism • Risk of misleading conclusions	• Hesitation in operational deployment • Increased regulatory scrutiny	• Hybrid validation combining in silico and empirical approaches • Conservative application
Interpretability and transparency	• Complex latent representations • Black-box model architectures	• Limited insight into learned biological mechanisms • Reduced explainability	• Lower stakeholder trust • Challenges in regulatory approval	• Explainable AI techniques • Model documentation and auditing
Ethical and legal considerations	• Ownership of genomic data • Use of AI-generated biological designs	• Constraints on data use and sharing • Unclear responsibility for outcomes	• Slower adoption • Societal resistance to AI-driven breeding	• Development of ethical guidelines • Clear regulatory frameworks
Long-term and multigenerational effects	• Limited ability to simulate long-term genetic consequences • Delayed phenotypic outcomes	• Incomplete assessment of cumulative effects • Uncertain predictions	• Risk to sustainability and animal welfare • Difficulty in strategic planning	• Longitudinal modeling • Integration of evolutionary principles
Integration and deployment barriers	• Complexity of existing agricultural systems • Lack of interoperability	• Fragmented implementation • Reduced practical impact	• Limited return on technological investment • Operational inefficiencies	• System-level integration standards • Stakeholder training and engagement

### 5.1 Data challenges

The limitations on data used are one of the most universal constraints on the proper usage of generative AI in animal genomics. Generative models also rely on the learning of correct data distributions and latent structure, and thus are highly sensitive to the failure of both data quality, representativeness, and integration. The biological diversity, selection breeding, and unequal research funding influence the livestock genomic datasets and generate a data environment that is highly diverse and unequal. These features make the model training and assessment harder, as well as have a direct effect on the biological feasibility and generalizability of the generative outputs in smart agricultural systems (Venegas et al., 2025; Zhu et al., 2025).

The main problem is that data is not equally available in terms of species, breeds, and production systems. The genomic resources are typically pooled in highly managed and economically powerful livestock groups, whereas numerous local, indigenous, or minor breeds are poorly characterized. This bias occurs because of historical and economic priorities and not due to biological relevance, but generative AI models that are trained on this data are bound to inherit these biases. Learned distributions can be skewed or narrow, and, in this case, generative models cannot capture the full range of genetic diversity that can support resilient and inclusive smart agriculture, which can be more problematic in larger and less studied groups (Babu et al., 2025; Rochus et al., 2025).

Compounding the problem of data sparsity is the integration of phenotypic, environmental, and management data with the genomic data. Interesting phenotypic traits are also often incomplete, irregularly recorded, and/or subject to context-specific factors, resulting in fragmented and noisy data. Although, in theory, missing data can be dealt with using generative models with the application of probabilistic inference, the effectiveness of the models relies on the presence of adequate contextual data. When sparsity and systematic bias interact, the models can be trained to acquire distorted representations, representing how data is collected rather than what biological relations do, which can lead to a lack of confidence in downstream uses (Bowler et al., 2024).

The heterogeneity of data standards and annotation also makes generative modeling more difficult. Various platforms and protocols generate livestock genomics and phenotyping data, and they may not be harmonized in metadata and commonly defined. Additional variability in resolution and reliability is added by environmental and sensor-derived data. The models of generative AI that are meant to combine such heterogeneous sources need to address the discrepancy between the modalities, which is technically difficult and can lead to spurious correlations. With smart agriculture systems based on built-in decision pipelines, these inconsistencies may spread through models and worsen robustness (Guerra-Manzanares et al., 2025).

There are other challenges entailed by population structure and relatedness that are inherent in the livestock breeding programs. Strong genetic correlations between individuals and generations have assumptions of independence and may cause generative models to fit artifacts of a population and not generalizable biological laws. Synthetic data generation and variant modeling can increase or decrease genetic diversity without valid reasoning, instead of enhancing long-term adaptability without care. The constraint is especially consequential to smart agriculture, where diversity is the key to being resilient in the face of environmental change (Szatkownik et al., 2024).

Additional complexity is brought about by temporal heterogeneity. Livestock data tend to cover extended durations of time where breeding goals, management methods, and environmental circumstances alter. The generative models learned with temporally mixed data can confuse old patterns with the existing biological relationships and lose their significance in the decision-making of modern times. To handle temporal dynamics, it is necessary to explicitly model time-dependent influences and ensure their consideration, but these methods are currently not easy to implement in practice (Kim et al., 2024).

Generative AI is also adversely affected by data quality and noise. There is uncertainty created by sequencing errors, genotyping errors, and non-uniform phenotype measurements, which may distort biological signals. Even though generative models can express the uncertainty on a probability basis, too much noise may drown out meaningful patterns. In agro-environmental settings where cost-related factors may sometimes restrict the quality of data, the separation of biological variation and technical artifact has remained a persistent problem (Jobin et al., 2024).

In general, the policy of data use and access determines the generative uses of AI. Ownership of data and fragmentation of proprietary restrictions create barriers to access to different datasets, restricting the development and evaluation of models. Although synthetic generation of data has potential avenues of alleviating access barriers, the representativeness of the synthetic data is still conditional on the underlying data. The inability to make generative AI useful in animal genomics and smart agriculture is limited without structured and harmonized data ecosystems.

### 5.2 Ethical, legal, and social considerations

The most important aspect of the responsible use of generative AI in animal genomics is ethical, legal, and social because these technologies do not affect the technical results only, but also the values of society, the nature of governance, and the level of trust these technologies develop. These considerations are therefore critical to the responsible development and deployment of generative AI in animal genomics, extending beyond technical challenges. The practical aspect of the integration of generative models in livestock systems intensifies the impact of genomic decision-making, which creates the issue of accountability, legitimacy, and long-term custodianship. These considerations should be taken into account to ensure that smart agriculture develops in ways that conform to the expectations of society and the ethics of its work (Janssen, 2025).

Animal welfare is one of the main ethical issues. Genetic interventions and management plans can be advanced using generative AI and become more accurate, yet they involve the risk of conceiving animals as primarily products of optimization instead of living beings. Although better genetic understanding could lead to better health and resilience, welfare trade-offs could be increased by poorly managed optimization goals. It is possible to have the abstraction of biological systems into computational models, which makes decision-makers less likely to think about ethical issues unless welfare considerations are directly integrated into modeling frameworks and decision criteria (Ghavi Hossein-Zadeh, 2025).

Further associated with welfare is the moral obligation to maintain genetic diversity and long-term flexibility. The use of generative models with a strong emphasis on narrow goals can unintentionally enhance genetic homogenization, compromising resilience and making a person susceptible to new stress factors. Ethical stewardship suggests striking a balance between a short-term efficiency approach and intergenerational accountability so that generative AI will help maintain biodiversity and resilience in systems instead of undermining them (Mirkute, 2025).

There are also legal issues associated with the ownership, access, and intellectual property of data, which complicate deployment. Genomic data has a high commercial value, and the capability of the generative AI to generate synthetic data and new biological constructs has left unanswered questions about ownership and rights over them. The current legal systems tend to be behind the technological capacities, causing a sense of uncertainty, which might slow down innovation or concentrate gains to those actors that have more legal and technical assets (Kalyvaki et al., 2025).

In non-human genomics, there are also privacy and confidentiality issues. Genomic information on livestock is strongly associated with breeding techniques and competitive advantages that are proprietary. Even without using explicit identifiers, the generative models trained on sensitive data can unintentionally disclose valuable information. The importance of making sure that privacy-conserving solutions are efficient is to be very cautious when trying to evaluate them and clearly communicate their disadvantages (Xu et al., 2024).

Another very important dimension is social acceptance. Trust, cultural values, and historical experiences have shaped the perceptions of genetic technologies in food systems among the people. The uncertainty of generative AI can even increase the fear of inorganic intervention or the extinction of the old, especially in cases when the processes of decision-making are not open. Relevant consultation with interested parties and effective communication of objectives and protection are thus critical in ensuring social legitimacy (Baumann et al., 2025).

The outcomes of ethics are also determined by equity and access. Even sophisticated generative AI tools may demand extensive resources and knowledge, and small-scale producers and resource-constrained areas may be limited in their ability to produce them. When the large industrial activities are the main beneficiaries, generative AI will create more inequalities and not help in promoting inclusive smart farming. To apply ethics, one must be concerned with accessibility, capacity building, and flexibility in different contexts (Nakagomi et al., 2025).

Ethical principles are transparency and accountability. The generative model becomes complicated and hides the decision pathways, making it difficult to attribute responsibility to the results. Stakeholders should be in a position to question and criticize AI-based recommendations in the agricultural setting, where choices have an impact on welfare, livelihood, and sustainability. It is therefore necessary to establish governance mechanisms that promote accountability (Thirunagalingam, 2024).

The regulatory frameworks also pose some other challenges, because the current policies might not be capable of dealing with the capabilities of generative AI. Innovation and oversight need to have a balance where a governance system is adaptive and therefore changes with technology. Finally, ethical, legal, and social considerations should not be considered as a static problem, but instead as a dynamic one that should be addressed throughout the research and implementation of deployments, but not in a retrospective manner (Mansouri et al., 2025).

### 5.3 Model generalization and bias

The essential constraints to the reliability and equity of generative AI in animal genomics are model generalization and bias. Generative models are intended to be able to extrapolate beyond the observed data, but whether they can generalize meaningfully is dependent on the diversity and representativeness of the training data. In livestock systems where population structure is strong and the environment is highly heterogeneous, robust generalization is especially difficult to attain. Selective breeding causes population stratification, and thus generative models are learned that can be particular to a breed or management system. Such models can give faulty results when used outside these contexts. This is further enhanced by bias in training data, in which the historical priorities of data collection can be modeled and reinforced by generative models generating synthetic output and predictions in a manner that undermines sustainability or equity objectives (Mpampi, 2025; Pérez-Enciso et al., 2025). Another generalization challenge is the heterogeneity of the environment. Genotype-environment interactions are the core of livestock phenotypes, but environmental data are usually poorly represented or poorly incorporated into the models. Models that are trained under restricted settings can thus fail to capture local conditions and hence become less useful in other systems involving agriculture (Bussiman et al., 2025).

The predictive power of generative models enhances the chances of overfitting, especially in high-dimensional genomic data with high correlations. Models can acquire ad hoc spurious relationships that are plausible but biologically unfounded. Generalization is also not easily evaluated since ground truth is frequently not accessible to novel or synthetic outputs. Uncertainty also represents bias. The confidence of generative models can be high in areas of data space that are poorly represented, which leads to excessive reliance on the prediction. To deal with this, we should have technical methods of quantifying uncertainty and cultural changes in the interpretation of outputs (Morales et al., 2025).

Interpretability is important in the diagnosis of failures of bias and generalizations. Knowledge of what properties are motivating model behavior allows one to be able to differentiate between biological insight and artifact. The technical solutions require governance mechanisms, transparent documentation, and regular monitoring as complements to inclusive and responsible smart agriculture by generative AI (Ramachandram et al., 2025).

### 5.4 Computational and resource limitations

The use of generative AI in animal genomics is greatly constrained by computational and resource constraints, which also influence the equity of using AI in animal genomics. Genomic data of high dimensions and highly complex generative models require large amounts of computing resources, storage, and specialized knowledge. Such requirements can be too large to allow many agricultural stakeholders to participate or scale up. Data management and storage also increase the burden on infrastructure, especially when incorporating continuous phenotypic and environmental data. The energy usage of large-scale model training poses sustainability challenges, and there are conflicts between computational intensity and sustainability (Robben, 2025). Human expertise is also another limitation. Generative modelling is an interdisciplinary task, with unevenly distributed skills required to develop and maintain it. These issues are made worse by infrastructure disparities that threaten to create digital gaps in the case of smart agriculture (Avila et al., 2025).

There are sustained resource requirements with deployment and maintenance since models need to change with changing biological and environmental conditions. Generative models can be outdated without a long-term investment. To solve these shortcomings, the development of model efficiency, shared infrastructure, and capacity building is necessary to make sure the generative AI technologies are fairly accessible (Thakare, 2025).

Within computational constraints, an additional consideration is the environmental and energy footprint associated with training and deploying large-scale generative models. Developing large foundation models for biological data often requires substantial computational resources, such as high-performance hardware and prolonged training times. This can result in significant energy consumption. This raises concerns about the carbon footprint of these approaches, especially when scaled across multiple training iterations or applied in resource-intensive research environments. These challenges are particularly relevant in smart agriculture, where sustainability is a primary goal and technological solutions are expected to promote, rather than hinder, environmental efficiency. To address this issue, we need to develop more energy-efficient model architectures, optimization strategies, and deployment frameworks. We also need to pay more attention to the trade-offs between model complexity, performance, and environmental impact (Malik, 2025).

### 5.5 Validation and interpretability

Validation and interpretability play a key role in the responsible generation of AI use in animal genomics, since both will dictate whether the results can be relied upon and action taken. Generative tasks typically do not have a clear sense of ground truth, and one needs to prove them based on biological plausibility, robustness, and downstream usefulness (Feldman & Benoliel, 2025).

The domain knowledge and collaboration in interdisciplinary work are required to ensure that there is biological validity. Strength in more than one population and setting has to be evaluated to prevent failure in particular settings. The useful, though not complete, validation is given by downstream task performance because the enhancement could be hiding more profound levels of bias or constraint (Senoner et al., 2024). Interpretability is necessary in how the models behave and to foster trust. Latent representations of complex latent models are frequently used to generate models that are hard to correlate with biological codes. These representations must be made meaningful in order to make prudent decisions in smart agriculture (Ge et al., 2025).

Additional complexity is introduced by probabilistic outputs, which will need intuitive uncertain communication. Multimodal integration also makes validation and interpretability more difficult because the errors can carry over to data layers. The lack of standard benchmarks obstructs the systematic assessment, which explains the necessity of community-based standards (Feldman & Benoliel, 2025).

Ethical accountability overlaps with validation and interpretability because opaque models conceal the accountability of the results. To overcome these issues, it is necessary to resort to methodological innovation, open governance, and continued assessment. The process of validation must be seen as a process of continuous development in line with the dynamism of smart agro systems, rather than a one-time check (Mohammed, 2025).

A critical and increasingly recognized challenge in generative modeling for genomics is biological hallucination. This occurs when models generate sequences that appear statistically or structurally plausible but lack functional validity in biological systems. Although generative frameworks can produce novel promoter, regulatory, or protein sequences that conform to learned patterns, these sequences do not inherently exhibit the intended biochemical activity, such as binding to specific transcription factors or maintaining stable structural conformations. This limitation reflects a fundamental gap between sequence-level modeling and functional biological realization. Addressing this challenge requires integrating post-generation validation strategies, such as in silico filtering approaches that assess structural stability, binding affinity, and regulatory potential before experimental testing (Calvanese et al., 2025). Incorporating predictive modeling tools for protein folding, molecular interactions, and regulatory dynamics can serve as an intermediate validation layer, reducing the risk of propagating nonfunctional or misleading designs. Ultimately, overcoming biological hallucination depends on developing hybrid frameworks that combine generative modeling with mechanistic constraints and multi-level validation pipelines. These frameworks ensure that generated outputs are statistically coherent and biologically meaningful (Wanqing et al., 2025).

## 6 Future prospects and research directions

Despite the rapid progress of generative modeling, translating these approaches into livestock systems is still in its early stages. Many of the architectures discussed in the literature were developed and validated in human genomics or general computational settings. These settings differ substantially from those in animal agriculture with regard to data availability and experimental frameworks. Therefore, their direct applicability to livestock genomics requires careful evaluation, including validation under species-specific biological constraints and production environments. Bridging this gap is essential for transitioning from conceptual innovation to practical implementation in smart agricultural systems (Patil et al., 2023). The further evolution of generative AI in animal genomics is highly connected to the further evolution of smart agriculture, which aims to develop into systems that are more adaptive, predictive, and sustainability-oriented. With the development of generative models into exploratory analytical engines as well as constituents of integrated decision-making infrastructures, the research agenda has increasingly focused on responsibility in their deployment (along with methodological novelty), system-level integration, and the long-term biological and social consequences. The guidelines in this section are symptomatic of the transition of proof-of-concept applications to scalable, powerful, and ethically sound models that can be used in the next generation of livestock systems. All these directions outline the opportunities of using generative AI to go beyond descriptive and predictive paradigms in animal genomics to anticipatory, design-oriented, and systems-based models that can be characterized as consistent with the aims of smart agriculture (Eckhardt et al., 2025; Paul & Machavaram, 2025). Future research should explore strategies for transferring learning to enable the adaptation of generative models from data-rich livestock populations to underrepresented breeds and species. Future research directions for generative AI in animal genomics and smart agriculture are listed in Table 16. Also, the technology readiness levels of generative AI applications in animal genomics are presented in Table 17. To distinguish between conceptual advances and empirically validated applications more clearly, the current generative AI landscape in animal genomics can be evaluated in terms of technology readiness. While some approaches have begun to demonstrate applicability in livestock systems, many remain in the early conceptual stages and often originate from human genomics or general computational research. Evaluating these developments through the lens of technology readiness clarifies their current maturity, limitations, and pathways toward practical implementation in smart agriculture.

**Table 16:** Future research directions for generative AI in animal genomics and smart agriculture.

Research direction	Scientific focus	Role of generative AI	Expected impact on animal genomics	Relevance for smart agriculture
Generative AI-driven genome engineering	• Rational design of genomic modifications • Functional optimization of genetic elements	• Generates candidate genomic edits under biological constraints • Simulates downstream genomic effects	• Improves precision and efficiency of genome engineering • Reduces trial-and-error experimentation	• Enables targeted and sustainable genetic improvement • Supports long-term system resilience
Multimodal generative modeling	• Integration of genomic, phenotypic, environmental, and sensor data • System-level biological complexity	• Learns joint representations across heterogeneous data streams • Generates coherent multimodal predictions	• Enhances understanding of genotype-phenotype-environment interactions • Improves trait modeling	• Enables holistic farm-level intelligence • Supports adaptive and context-aware management
Federated and privacy-preserving genomics	• Distributed genomic datasets • Data ownership and confidentiality	• Trains generative models across decentralized data sources • Generates privacy-preserving representations	• Expands access to diverse genomic resources • Reduces data-sharing barriers	• Facilitates collaborative smart agriculture ecosystems • Builds trust among stakeholders
Rare breed and conservation genomics	• Underrepresented and endangered livestock populations • Genetic diversity preservation	• Generates synthetic data to augment sparse datasets • Transfers knowledge from related populations	• Supports genomic characterization of rare breeds • Aids conservation strategies	• Promotes biodiversity and resilience • Aligns productivity with conservation goals
Real-time integration with smart farming tools	• Continuous data streams from sensors and management systems • Dynamic decision contexts	• Generates real-time predictions and simulations • Updates models adaptively	• Enables responsive genomic analysis • Improves temporal relevance of predictions	• Supports precision and automation in farm operations • Enhances decision timeliness
Digital twins development for livestock	• Virtual representation of animal genomic and phenotypic states • Longitudinal system behavior	• Simulates future biological trajectories • Explores management and breeding scenarios	• Improves prediction of long-term genomic outcomes • Enhances system understanding	• Enables proactive planning • Supports sustainability and risk management
Explainable and interpretable generative models	• Transparency of AI decision processes • Biological interpretability	• Develops interpretable latent representations • Generates explanations alongside outputs	• Increases trust in AI-derived genomic insights • Facilitates biological validation	• Improves stakeholder acceptance • Supports regulatory compliance
Bias-aware and fair generative modeling	• Representation of diverse breeds, systems, and environments • Equity considerations	• Detects and mitigates bias during data generation • Produces balanced synthetic outputs	• Improves generalization across populations • Protects genetic diversity	• Supports inclusive and responsible smart agriculture practices
Regulatory frameworks and responsible AI	• Ethical, legal, and social dimensions of AI-driven genomics • Governance structures	• Embeds constraints and accountability mechanisms into model design • Supports auditability	• Ensures responsible use of generative genomics • Aligns innovation with societal values	• Facilitates adoption and long-term legitimacy of smart agriculture technologies
Sustainability-oriented generative modeling	• Long-term environmental, welfare, and genetic outcomes • Systems sustainability	• Simulates long-term impacts of genomic and management decisions • Explores trade-offs	• Aligns genomic innovation with sustainability goals • Supports resilient breeding strategies	• Guides sustainable resource use • Balances productivity with environmental stewardship

**Table 17:** Technology readiness levels of generative AI applications in animal genomics.

Application domain	Generative AI approach	Primary origin of development	Level of empirical validation in livestock	Current technology readiness	Key limitations and gaps
Data augmentation and synthetic genomic data	GANs, VAEs, and diffusion models	• Computer science • Human genomics	• Limited livestock-specific validation • Mostly simulation-based studies	• Emerging to early applied	• Generalization across breeds • Validation under real-world conditions
Variant prediction and discovery	Sequence-based generative models	• Human genomics • Computational biology	• Early-stage application in livestock • Indirect validation via comparative genomics	• Emerging	• Species-specific regulatory complexity • Limited labeled datasets
Protein and gene sequence design	Transformer-based generative models	• Protein engineering • Human biomedical research	• Minimal direct livestock validation • Mostly conceptual or translational	• Conceptual to early experimental	• Functional validation in vivo • Biological safety and regulatory concerns
Genomic selection and trait prediction	Hybrid generative–predictive models	• Statistical genetics • Machine learning	• Moderate validation in livestock contexts • Extensions of established models	• Early applied	• Integration with breeding programs • Interpretability and robustness
Gene regulation and epigenomics	Generative sequence and regulatory models	• Functional genomics • Human epigenomics	• Limited livestock-specific studies • Cross-species inference	• Emerging	• Sparse epigenomic data in livestock • Tissue-specific variability
Precision breeding and reproductive technologies	Generative imaging and simulation models	• Computer vision • Reproductive biology	• Early-stage validation in controlled settings	• Emerging to early applied	• Standardization of phenotyping • Integration with genomic pipelines
Cross-species genomic modeling	Transfer learning and generative models	• Comparative genomics • AI research	• Indirect validation via cross-species transfer	• Emerging	• Domain adaptation challenges • Evolutionary divergence effects
Digital twins and integrated systems	Multimodal generative models	• Systems biology • Smart agriculture technologies	• Conceptual to limited pilot implementations	• Conceptual to emerging	• Real-time integration • Infrastructure and scalability constraints

### 6.1 Generative AI-driven genome engineering

One of the most radical future directions, where generative AI and animal genomics converge, is genome engineering being driven by Generative AI. Over the years, genome engineering has been a field of incremental innovation with an empirical experimental approach to identifying and testing hypotheses, which appears less and less generous as biological complexity and the space of potential genetic interventions continue to grow. With a radically new paradigm, generative AI can be used to explore the space of genomic designs informed by learned representations of biological structure, function, and constraint. Generative models can be used to enable systematic reasoning about genetic modification by modeling the genomic sequences and regulatory architectures as probabilistic systems, instead of using trial-based refinement (DaSilva et al., 2025).

In the context of animal genomics, this has some important implications since genetic interventions are interrelated with complex physiological processes, management systems, and environmental conditions, on long time scales. Genome engineering with the help of Generative AI has the potential to predict downstream consequences of genomic alterations by modeling the spread of modifications across regulatory networks and phenotypic outcomes. This foresight ability brings genome engineering nearer to the concept of smart agriculture, in which the result will not just be assessed based on its short-term benefits, but also its long-term effects on productivity, livelihood, and survival (Pasupuleti, 2025; Robben, 2025).

An important area of future research is in the integration of genome engineering into a systems-based goal as opposed to the maximization of single components of genetics. Interactions between two or more genomic elements can be represented by generative models, which are able to promote the design of coordinated changes that capture trade-offs between biological functions. This kind of strategy will foster the move towards a process of narrow optimization to balanced practices that consider robustness, adaptability, and sustainability. This systems view is necessary in livestock systems where genetic transformations can have an impact on vast populations across generations; hence, responsible innovation is necessary (Yadav et al., 2024; Koluman et al., 2025).

Genome engineering based on Generative AI is also characterized by another vital feature, which is its ability to be refined repeatedly. Genome engineering strategies can be developed with scientific knowledge and agronomic conditions, as genomic, phenotypic, and environmental data are constant and can undergo constant updating with new information. This flexibility is opposed to the fixed design methods and is aligned with a learning-based model of innovation in line with the focus of smart agriculture on flexibility and responsiveness (Gakhar et al., 2025).

Although potentially promising, there are significant scientific and ethical issues associated with Generative AI-based genome engineering that define the research demands of the future. The biological validity is also an important concern since generative models can suggest genomic configurations that are statistically consistent but that are biologically impossible or unfavorable. The answer to this is more comprehensive exposure to mechanistic biological knowledge, as well as excellent validation structures. Ethical and governance issues are also more acute when the generative AI is taken to the design stage, with the key aspect of transparency, control, and alignment with the values of society (Jones, 2025).

### 6.2 Multimodal generative models

Multimodal generative models represent a key research direction in developing animal genomics in smart agriculture due to the growing accessibility and significance of non-homogeneous data streams. The contemporary livestock systems produce a variety of data that reflects both genetic, physiological, environmental, and managerial production aspects. Every modality can provide some understanding of biological processes, but none of the data types is adequate to elucidate complex traits and results. Multimodal generative AI aims to overcome this drawback by learning common representations in which information is combined across modalities, which allows for a much more complete modeling of livestock systems (Lee et al., 2025; Venegas et al., 2025).

The theoretical basis of multimodal generative models consists of the fact that the genotype-phenotype and environment are strongly interdependent and cannot be completely studied individually. Genomic data constitute biological potential, though phenotypic expression is brought about by regulatory mechanisms of environmental and management circumstances. Multimodal generative models are intended to model these dependencies by learning probabilistic dependencies between types of data with different structures and time series properties. Such a holistic approach can especially be helpful in the case of smart agriculture, where decision-making requires a dynamic interaction between several factors (Liu et al., 2025; Seebacher & Little, 2025).

Methodologically, multimodal generative models present the chance to solve the problem of data sparsity and imbalance, which animal genomics has long faced. Some data modalities are extensively distributed, and those that are restricted or are not collected uniformly. The generative frameworks can utilize common latent structures to deduce the missing information on modalities and can hence transfer the knowledge among data-rich and data-poor sources. It is capable of more inclusive and scalable implementations of advanced analytics in a variety of livestock systems (Khan et al., 2025; Robben, 2025).

Multimodal generative models also have a better predictive and exploratory capacity as they base their inferences on more than one source of evidence. Combining genomic, phenotypic, and contextual data, these models are able to produce predictions with biological complexity more accurately than those provided by single-modality methods. Their generative quality also facilitates the exploration of the scenario, whereby researchers and practitioners can investigate the impacts of alterations in one modality in the context of a particular genetic and environmental condition. This ability is extremely similar to the anticipatory and adaptive objectives of intelligent agriculture (Shamim et al., 2025).

Nevertheless, the generation of multimodal models of generation presents fresh challenges that characterize the critical areas of future research. Representation, synchronization, and quality control are important tasks to be undertaken when aligning heterogeneous data when modalities of interest vary in scale and time resolution to a significant degree. As the number of modalities grows, model complexity grows, and this brings up issues of computational efficiency, robustness, and interpretability. To cope with these obstacles, it will be necessary to make improvements that would not compromise the expressive power, but rather make it more transparent and practical, which will be aided by the introduction of biological knowledge into the design of the models (Patel, 2025).

### 6.3 Federated and privacy-preserving genomics

The role of federated and privacy-preserving genomics is a vital research path for making collaborative innovation possible in animal genomics without violating data ownership and confidentiality. The genomic data of livestock are typically dispersed between organizations and geographical boundaries, with access being limited by commercial, regulatory, and ethical factors. Such restrictions restrict the variety and size of datasets that could be used to train generative models, their generalizability, and their influence. The federated learning models present one way to eliminate these obstacles by allowing joint model training without requiring information to be shared centrally (Calvino et al., 2024).

Generative models are trained on decentralized data sources in a federated setting, and the updates on individual models are aggregated to enhance common representations. This will be a natural method of the livestock genomics ecosystem, in which data are naturally fragmented in breeding programs, farms, and institutions. In the case of smart agriculture, federated methods can be used to construct generative models based on varied genetic backgrounds and production settings that are more robust and do not interfere with the local control of data (Min et al., 2025).

Privacy protection methods also enhance the viability of group genomics by mitigating the chances of leakage of sensitive data. In the case of local raw data, generative models can still be informed by information regarding single datasets. Privacy-preserving techniques will help alleviate this risk, facilitating trust among stakeholders and making more people engage in data-driven agriculture. In animal genomics, in which data can be vital strategic assets, these protections can be critical to maintaining the partnership (Zafar et al., 2025).

Federated and privacy-preserving genomics also pose methodological issues that influence future research priorities. Distributed training has to face the heterogeneity in information quality, size, and distribution among participants, as well as the heterogeneity in computing resources. Generative models are required to learn the common biology but to tolerate local variation, and this is especially difficult in a livestock system with a strong population structure and a large environmental heterogeneity. It is still a topic of research to come up with algorithms that can withstand such heterogeneity (Sørensen & Sørensen, 2025).

In this context, several cryptographic and algorithmic approaches have emerged as promising solutions for securing sensitive genomic data in distributed learning environments. Techniques such as secure multiparty computation and homomorphic encryption allow for collaborative model training across multiple data holders without requiring direct data sharing. This preserves confidentiality while enabling joint analysis. Additionally, incorporating differential privacy mechanisms into model training limits the risk of re-identification from generated outputs or shared parameters. Recently, privacy-preserving federated learning frameworks have integrated these methods with decentralized optimization strategies, enabling secure and scalable learning across geographically distributed livestock datasets. Adopting these techniques is expected to be critical in balancing data utility, ownership, and privacy in future genomic applications (Aherrahrou et al., 2024; Kalejaiye et al., 2025).

In a more general sense, federated and privacy-sensitive solutions also have significant implications for equity and inclusion in smart agriculture. They can facilitate smaller and resource-constrained actors to both contribute to and benefit from the state of advanced generative models by reducing the participation barriers. This inclusion allows for the development of agricultural intelligence, which is more globally relevant and robust to develop. Meanwhile, there should be a careful balance between privacy protection and model performance, especially in cases where biological accuracy and interpretability are important in the application (Tahir et al., 2025).

### 6.4 Generative AI for rare breeds and conservation

Generative AI applied to rare breeds and conservation is a potential future path that will shift technological innovation towards biodiversity preservation and sustainability goals. The rare and adaptively evolved breeds carry with them genetic diversity, ecological balance, and cultural heritage, which is, however, underrepresented in genomic studies because of the limited size and availability of data. Generative AI has methodological flexibility that may be used to overcome these limitations by learning with limited information and transferring knowledge between similar populations (Tăpăloagă et al., 2025).

An important input of generative AI in this regard is that it can be used to model genetic variation when there is a lack of data. It can be possible to make better and more informed judgments regarding diversity, inbreeding vulnerability, and adaptive capacity of rare populations with the help of generative models that learn latent representations reflecting underlying biological organization. It is especially useful in conservation-driven agriculture, in which choices need to be made between genetic identity preservation and adjustment to evolving circumstances (Shaikh et al., 2025).

The scenario-based exploration is also well-supported by generative AI and is particularly appropriate to conservation planning. Simulation data and artificial results allow testing alternative plan implementation without imposing further strain on small populations. Such in silico exploration is in line with the precautionary principles on which conservation is guided to ensure that stakeholders can predict the consequences that may be likely to be caused by the implementation of interventions. Such capabilities are particularly pertinent in systems of smart agriculture that attempt to combine conservation and production goals (Pérez-Enciso et al., 2025).

On top of technical reasons, there are valuable ethical issues related to the use of generative AI on rare breeds. Efficiency is not the only basis in conservation, but other values in conservation are associated with heritage, identity, and intergenerational responsibility. Generative models should thus be deployed in a manner that does not violate such values and does not unwillingly homogenize or erase particular characteristics. Close interaction with conservation principles and stakeholders would be necessary in responsible research in this area (Laine et al., 2025).

As a methodological approach, rare-breed applications can serve as a useful testing ground to develop generative models, which can work with data constraints. The future progress in this field can be generalized to other situations where the data are sparse or heterogeneous, and the concept of conservation-oriented studies can be further applied to smart agriculture.

### 6.5 Integration with real-time smart farming tools

Combining the use of generative AI and real-time smart farming devices is an important move towards ensuring that the gains in animal genomics transform into functional veterinary systems. Smart agricultural technologies produce a continuous data stream that shows the dynamics of the environment and animal state, and genomic data give a consistent understanding of biological possibilities. These layers can be connected by generative AI, enabling context-aware and predictive systems (Subeesh & Chauhan, 2026).

One of the key opportunities is to apply generative models to explain the real-time observations with reference to the genomic predispositions. By comprehending the relationship between genetic background and responses to environmental and management conditions, generative AI can be used to distinguish transient variation and patterns that may be considered biologically significant. Such integration makes it possible to make decisions much more precisely and individually, which increases the applicability of smart farming interventions (Min et al., 2025; Mirkute, 2025).

Generative AI also makes real-time systems shift to adaptive and anticipatory strategies instead of a rule-based response. Generative models enable a probabilistic representation of uncertainty and variability, which assists in detecting any emerging problems early and testing other possible interventions. Incorporating these features into intelligent agricultural devices is compatible with the vision of agriculture as a system with learning processes as a continuous process (Sushmitha et al., 2025).

In livestock systems, digital twins are evolving from data-integrative representations into more autonomous and predictive systems. In the near future, digital twins are expected to primarily serve as integrative platforms, combining environmental, phenotypic, and genomic data to facilitate scenario analysis and decision-making. In the long term, autonomous systems that can continuously mimic biological processes and optimize breeding and management techniques without direct human intervention may be created, thanks to developments in generative modeling, real-time data assimilation, and adaptive learning. The technological, ethical, and regulatory requirements vary significantly between these stages of development. Thus, this progression emphasizes the importance of distinguishing between current implementations and future visions (Arulmozhi et al., 2024; Ghavi Hossein-Zadeh, 2025). Table 18 illustrates the evolution of digital twins in livestock systems.

**Table 18:** Evolution of digital twins in livestock systems: Near-term and long-term scenarios.

Dimension	Near-term horizon (approximate short-term development)	Long-term horizon (autonomous and fully integrated systems)
Conceptual role	• Decision-support system • Data integration platform	• Autonomous system representation • Continuous system optimization
Data integration	• Combines genomic, phenotypic, and environmental data • Periodic updates	• Real-time multi-omics and sensor integration • Continuous data assimilation
Modeling approach	• Predictive and simulation-based models • Semi-static frameworks	• Fully generative and adaptive models • Self-updating dynamic systems
Decision-making role	• Supports human decision-making • Scenario evaluation	• Partially or fully autonomous decision-making • Real-time optimization
Use in breeding	• Assists in genomic selection and planning • Evaluates breeding strategies	• Autonomous breeding optimization • Continuous refinement of genetic programs
System adaptability	• Limited adaptability to new conditions • Requires recalibration	• High adaptability • Learns continuously from new data streams
Integration with smart farming	• Linked to precision livestock farming tools • Moderate interoperability	• Fully embedded in smart farming ecosystems • Seamless system-wide integration
Ethical and governance needs	• Focus on data ownership and transparency	• Emphasis on autonomy, accountability, and regulatory oversight
Technological maturity	• Emerging and partially implemented	• Conceptual to early developmental stage

Advances in edge computing and distributed learning paradigms are closely related to the practical application of generative AI in precision livestock farming systems. Centralized data processing is often impractical in agricultural settings with poor connectivity and limited infrastructure. Edge AI techniques enable local data processing directly on farm-based devices, lowering latency and eliminating reliance on constant network access (Avanija et al., 2024). Federated learning frameworks, on the other hand, support data privacy and operational resilience by providing a way to train models cooperatively across dispersed nodes without requiring direct data sharing. However, despite their theoretical benefits, these methods have several drawbacks in livestock environments. These include limited edge computational resources, inconsistent data quality across dispersed systems, and difficulty preserving model consistency due to sporadic connectivity. Furthermore, generative models are sensitive to data heterogeneity and have high computational requirements; therefore, integrating them into such decentralized architectures adds complexity. To overcome these obstacles, it will be necessary to develop more effective model architectures, flexible training methods, and reliable communication protocols suited to agricultural settings. The variability of farm-level infrastructure and the reliability of communication networks also impact the efficacy of federated and edge-based approaches (Palvancha et al., 2025; Thakur et al., 2025).

Emerging advancements in real-time, edge-based generative AI enable on-site data processing and decision-making, expanding the potential of smart livestock systems by eliminating dependence on centralized cloud infrastructure. To facilitate adaptive, context-aware management, these approaches envision generative models that function directly within farm environments and continuously integrate genomic, phenotypic, and environmental data streams. Though this paradigm is still in its early stages of development, it signifies a move toward more resilient and autonomous agricultural systems, especially in environments with poor connectivity. While achieving this goal requires improvements in hardware integration, robust decentralized learning frameworks, and model efficiency, it points to a crucial path for the future development of generative AI in smart agriculture (Santhosh, 2024; Yu et al., 2025).

Future research interests in this field are influenced by technical issues. The real-time integration should also have effective models that can work within the constraints of latency and reliability. The heterogeneous platform interoperability also implies the standard representation and modular design. The response to these issues is critical in the translation of generative AI in analytical settings to a working smart agriculture system.

### 6.6 Regulatory frameworks and responsible AI

Responsible AI and regulatory frameworks represent a research pathway that forms the basis of ensuring that the application of generative AI to animal genomics and smart agriculture is trustworthy and socially acceptable. Regulatory systems need to change to manage their probabilistic, adaptive, and opaque nature as generative models are increasingly used to make breeding and management decisions. The era of traditional regulation methods might not be appropriate in terms of technologies that evolve and adapt with time, so new types of regulation must be learned. Responsible AI principles offer principles for such a development by highlighting transparency, accountability, fairness, and robustness. In the context of animal genomics, responsible AI would mean that the design of gen-models must have a clear purpose, assumptions that must be documented, and performance and unintended consequence monitoring mechanisms. These kinds of practices ensure regulatory compliance and the trust of the people (Thirunagalingam, 2024; Chen et al., 2025).

One of the areas of intersection between regulation and responsible AI is data governance. To balance between data sharing to innovate and the protection of sensitive information, it is essential to have clear structures that establish rights and responsibilities. The ultimate technologies in smart agriculture need a fair distribution of benefits and acknowledgment of the data contributors to ensure the continued existence of collaborative ecosystems (Shivpuja, 2025).

The international character of agriculture also makes the establishment of regulations more difficult, which is why similar standards and international cooperation should be considered. Generative AI facilitates the exchange of knowledge between regions faster, and the lack of homogeneous regulatory strategies becomes even more troublesome. Adjustable oversight systems that embrace learning systems and long-term impacts are thus a key concern in the research that should be done in the future. These future outlooks highlight the transformative nature of generative AI in the field of animal genomics as well as the circumstances under which such technology may be implemented responsibly and effectively in the smart farming system (Ampatzidis et al., 2025).

## 7 Conclusions

Though generative AI is still emerging rather than fully mature, it is reshaping the conceptual underpinnings of animal genomics within smart agriculture. While these methods offer new ways to simulate intricate biological systems and integrate different data sources, the practical application of these methods in livestock contexts is limited by biological complexity, data constraints, and the need for thorough validation. Integrating computational techniques with domain-specific knowledge and actual agricultural conditions will be crucial for advancing this field, as will improving model design. Closing the gap between theoretical innovation and practical implementation will be a major challenge in the future. This requires scalable solutions that can operate in the diverse, resource-constrained environments typical of livestock production and enhanced multimodal frameworks that link genomic data with phenotypic expression and management systems. However, to ensure reliability and trustworthiness, the limitations of generative models must be specifically addressed, such as issues with generalization, interpretability, and biological plausibility. Ultimately, generative AI should be viewed as a supplementary tool that enhances, rather than replaces, well-established animal genomics techniques. The long-term effects will be determined by interdisciplinary cooperation, ethical governance, and a persistent emphasis on validation and applicability. Generative AI has the potential to support livestock systems that are more adaptive, efficient, and sustainable. These systems would have a smaller environmental footprint and be more resource-efficient in producing animal-derived protein by aligning technological innovation with biological and agricultural realities.

## Ethical approval

This article does not contain any studies with human participants performed by any of the authors.

## Funding information

This research received no funding.

## Ethics statement

Not applicable: This manuscript does not include human or animal research.

## Data availability

Not applicable.

## CRediT authorship contribution statement

**Navid Ghavi Hossein-Zadeh:** Writing – review & editing, Writing – original draft, Visualization, Validation, Supervision, Project administration, Investigation, Conceptualization.

## Declaration of competing interest

The authors declare that they have no known competing financial interests or personal relationships that could have appeared to influence the work reported in this paper.
